# Structure of a diatom photosystem II supercomplex containing a member of Lhcx family and dimeric FCPII

**DOI:** 10.1126/sciadv.adi8446

**Published:** 2023-10-25

**Authors:** Yue Feng, Zhenhua Li, Xiaoyi Li, Lili Shen, Xueyang Liu, Cuicui Zhou, Jinyang Zhang, Min Sang, Guangye Han, Wenqiang Yang, Tingyun Kuang, Wenda Wang, Jian-Ren Shen

**Affiliations:** ^1^ Photosynthesis Research Center, Key Laboratory of Photobiology, Institute of Botany, Chinese Academy of Sciences, Beijing 100093, China.; ^2^ University of Chinese Academy of Sciences, Beijing 100049, China.; ^3^ China National Botanical Garden, Beijing 100093, China.; ^4^ Research Institute for Interdisciplinary Science, Graduate School of Natural Science and Technology, Okayama University, Okayama 700-8530, Japan.

## Abstract

Diatoms rely on fucoxanthin chlorophyll *a/c*-binding proteins (FCPs) for their great success in oceans, which have a great diversity in their pigment, protein compositions, and subunit organizations. We report a unique structure of photosystem II (PSII)–FCPII supercomplex from *Thalassiosira pseudonana* at 2.68-Å resolution by cryo–electron microscopy. FCPIIs within this PSII-FCPII supercomplex exist in dimers and monomers, and a homodimer and a heterodimer were found to bind to a PSII core. The FCPII homodimer is formed by Lhcf7 and associates with PSII through an Lhcx family antenna Lhcx6_1, whereas the heterodimer is formed by Lhcf6 and Lhcf11 and connects to the core together with an Lhcf5 monomer through Lhca2 monomer. An extended pigment network consisting of diatoxanthins, diadinoxanthins, fucoxanthins, and chlorophylls *a/c* is revealed, which functions in efficient light harvesting, energy transfer, and dissipation. These results provide a structural basis for revealing the energy transfer and dissipation mechanisms and also for the structural diversity of FCP antennas in diatoms.

## Teaser

A unique structure of PSII-FCPII from a diatom containing a member of the Lhcx family subunit and FCPII dimers was reported.

## INTRODUCTION

Photosynthetic organisms convert dispersed solar energy into stable chemical energy and store it in organic matters, which sustain almost all life forms on the earth. The light energy conversion reactions are performed by photosystem I (PSI) and photosystem II (PSII), which use light energy to drive charge separation and electron transfer reactions (*1*). Among them, the PSII core complex is associated with soluble phycobilisomes in prokaryotic cyanobacteria to harvest the dispersed light energy (*2*, *3*), whereas in eukaryotic algae and higher plants, it is associated with various trans-membrane light-harvesting protein complexes (LHCII). The number, sequences of LHCIIs, and pigments bound to LHCIIs differ among different organisms (*4*, *5*).

Structure of the PSII core has been solved from a cyanobacterium at a high resolution (*6*, *7*) and from red algae (*8*, *9*) by x-ray crystallography as well as cryo–electron microscopy (cryo-EM), which showed the arrangement and detailed structure of protein subunits within the PSII core. Structures of PSII-LHCII supercomplex from green algae and higher plants have also been solved by cryo-EM (*10*–*12*), which showed that LHCII are mainly organized into Lhcb monomers and trimers. The typical proteins of the Lhcb family mainly bind chlorophyll (Chl) *a*, Chl *b*, lutein, and some other carotenoids (*10*–*13*).

Diatoms are eukaryotic marine phytoplanktons that originate from red algae ancestors by secondary endosymbiotic events, which produce around 40% of the net primary productivity of the oceans or 20% of those on the earth (*14*, *15*). The light-harvesting antennas of the diatom PSII belong to the LHC family, but they bind Chl *a*/*c* instead of Chl *a/b*, and a large amount of fucoxanthin (Fx) instead of β-carotene (BCR), to harvest blue-green light (*16*, *17*); hence, they are called FCPs (fucoxanthin Chl *a/c* binding proteins). The xanthophyll cycle pigments for photoprotection in FCPs are diadinoxanthin (Ddx)/diatoxanthin (Dtx) instead of violaxanthin/zeaxanthin found in green algae and higher plants (*17*–*20*). Diatoms have much more *lhc* genes than higher plants, which are classified into three main groups, *lhcf*, *lhcr*, and *lhcx* (*21*–*23*). Lhcf proteins are the major antennas for PSII, whereas Lhcr proteins are mainly connected to PSI (*24*–*28*), and Lhcx proteins are considered to be mainly involved in photoprotection, as some of the Lhcx proteins have been found to function similarly to the LHCSR proteins in green algae (*21*, *28*, *29*). Different from PsbS (*30*), LHCSR proteins bind Chls and carotenoids that are associated with the xanthophyll cycle (*31*, *32*), whereas whether Lhcx proteins bind pigments is not known at present.

The structure of an isolated dimeric FCP from the pennate diatom *Phaeodactylum tricornutum* (Pt) has been solved by x-ray crystallography at a high resolution (*33*). On the other hand, FCP trimers were found in two centric diatoms *Cyclotella meneghiniana* and *Thalassiosira pseudonana* (Tp) by electron microscopy and cryo–electron tomography (cryo-ET) structural analysis (*34*, *35*). In addition, the structure of a PSII-FCPII supercomplex of the centric diatom *Chaetoceros gracilis* (Cg) has been solved by cryo-EM, which demonstrated the association of tetrameric FCP with the PSII core (*24*, *25*, *36*). These results demonstrate a high variety of protein organizations and pigment compositions among FCPIIs, which are largely different from those in PSII-LHCII of green algae and higher plants (*4*, *5*). This may constitute one of the major reasons for the success of diatoms in green light harvesting, efficient energy transfer, and photoprotection.

The dimeric FCP structure has been found in isolated forms but not in complex with PSII or PSI. This imposes a question as to whether it exists in vivo. In this study, we purified a PSII-FCPII supercomplex from *T. pseudonana* and analyzed its structure by cryo-EM at 2.68-Å resolution. We found both homodimeric and heterodimeric FCPIIs as major antennas surrounding the PSII core, which is different from the antennas in higher plant PSII-LHCII and Cg-PSII-FCPII supercomplexes. In addition, a monomeric Lhcx family protein Lhcx6_1 was found in the cryo-EM structure of PSII-FCPII, which binds Chls and Fxs. These results reveal the detailed features of FCPII dimers and LHCX associated with the PSII core, providing a structural basis for elucidating the mechanisms of light harvesting, excitation energy transfer (EET), and energy dissipation in Tp-PSII-FCPII.

## RESULTS

### Overall structure and structure of the PSII core

**Fig. 1.**
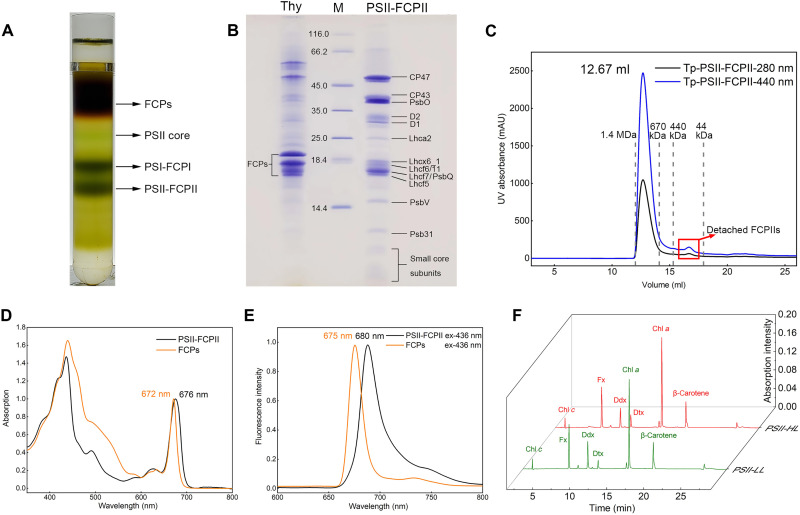
Sample preparation and characterization of PSII-FCPII of *T. pseudonana*. (**A**) Isolation of PSII-FCPII by sucrose density gradient centrifugation. The fourth band labeled as PSII-FCPII was collected and used for single particle analysis in this study. (**B**) SDS-PAGE analysis of the purified PSII-FCPII from *T. pseudonana*. Lane 1: Thylakoid membranes (5 μg Chl); lane 2: molecular weight marker (Thermo Fisher Scientific: 26610); lane 3: purified PSII-FCPII after size-exclusion chromatography (5 μg Chl). (**C**) Elution profile of the PSII-FCPII supercomplex and detached FCPIIs (marked by a red square) by size-exclusion chromatography (Cytiva, Superose 6 Increase 10/300 GL) at the absorbance of 280 and 440 nm, respectively. UV, ultraviolet. (**D**) Room temperature absorption spectrum of PSII-FCPII and FCPs. (**E**) Normalized 77 K fluorescence emission spectra of PSII-FCPII and FCPs excited at 436 nm. ex, excitation. (**F**) Analysis of the pigment composition of PSII-FCPII purified from cells grown under low light and high light by HPLC monitored at 445 nm and normalized based on the content of Chl *a*. Six major pigment peaks were identified; they are Chl *a,* Chl *c*, Fx, Ddx, Dtx, and BCR, respectively.

The PSII-FCPII supercomplex was purified from *T. pseudonana* (Fig. 1, A to C), in which six FCP subunits were identified from SDS–polyacrylamide gel electrophoresis (SDS-PAGE) and mass spectrometry (table S1, source data 1 to 8, and Fig. 1B). These FCP subunits are named Tp-Lhcf5-7, Tp-Lhcf11, Tp-Lhca2, and Tp-Lhcx6_1 based on the results of mass spectrometry (source data). Sequence analysis showed that Tp-Lhcf5-7 and Tp-Lhcf11 have high homology with each other as well as with Pt-Lhcf4 and Cg-Lhcf1, the FCP subunits forming a dimer in *P. tricornutum* and tetramer in *C. gracilis*, respectively, whereas Tp-Lhca2 and Tp-Lhcx6_1 belong to different clades (fig. S1). The absorption spectrum of the supercomplex showed a single peak at 676 nm (Fig. 1D), which is slightly longer than typical peaks of PSII core (673 to 674 nm), suggesting the presence of FCPIIs. The fluorescence spectrum showed a broad peak at 680 nm with an excitation wavelength of 436 nm (Fig. 1E), again reflecting the binding of a large amount of FCP to the PSII core. Pigment analysis showed that PSII-FCPII contains Chl *a*, Chl *c*, Fx, BCR, and some Ddxs and Dtxs, based on the results of high-performance liquid chromatography (HPLC) (Fig. 1F and table S2). The ratio of Ddx/Dtx is apparently higher in the sample prepared from cells grown under low light than that grown under high light, while other pigments do not change much between the two light conditions. This suggests that the Ddx/Dtx cycle becomes active under high-light conditions.

**Fig. 2.**
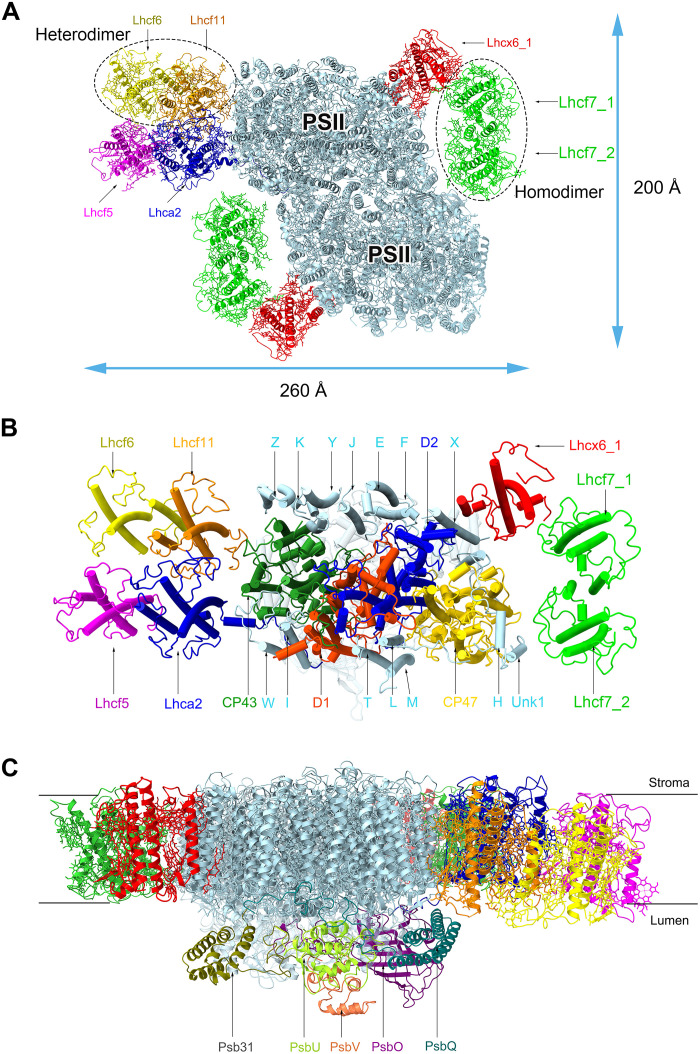
Overall structure of the PSII-FCPII supercomplex of *T. pseudonana*. (**A**) Overall structure of the PSII-FCPII supercomplex viewed along the membrane normal from the stromal side. (**B**) Structure of the larger monomer of Tp-PSII-FCPII viewed from the same direction as in (A), with all trans-membrane PSII core subunits and FCP subunits labeled. Small subunits of the PSII core were indicated with one letter. (**C**) Side view of the Tp-PSII-FCPII dimer structure with the five extrinsic proteins labeled, colors of the subunits are the same as in (A).

**Fig. 3.**
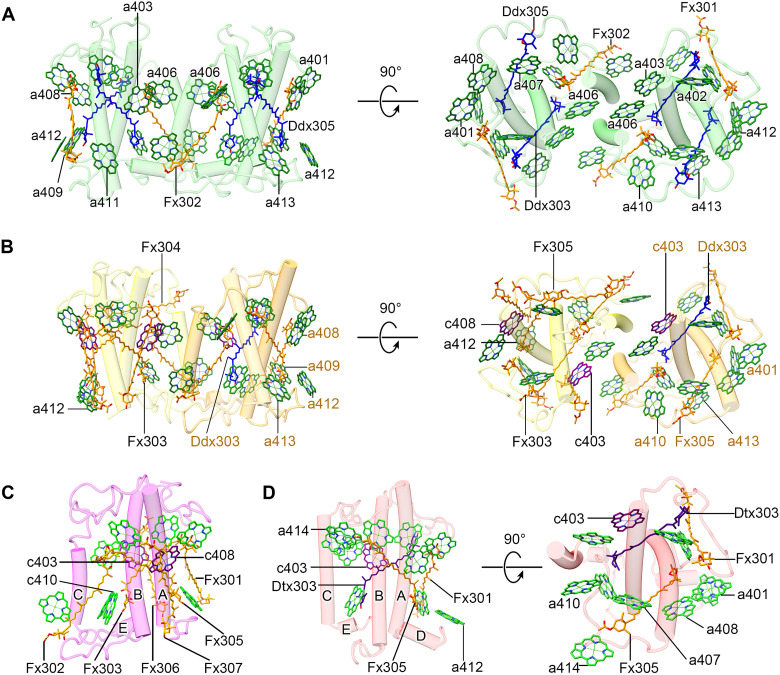
Structures and pigment arrangements in FCPs of the Tp-PSII-FCPII supercomplex. The pigments of Chl *a*, Chl *c*, Fx, Ddx, and Dtx are colored “green,” “purple,” “orange,” “blue,” and “indigo,” respectively, and shown in sticks. Only rings of the Chls are depicted, and their phytol chains are omitted. Several important pigment molecules are labeled. (**A**) Structure and pigment distribution of the Tp-Lhcf7 homodimer with a side view and a top view from the stromal side, respectively. (**B**) Structure and pigment distribution of the Tp-Lhcf6-Lhcf11 heterodimer. Lhcf6 is colored light yellow (left), and Lhcf11 is colored orange (right). (**C**) Structure and pigment distribution of Lhcf5. (**D**) Structure and pigment distribution of Lhcx6_1.

The isolated Tp-PSII-FCPII supercomplex was imaged by cryo-EM at 300 kV, and a total of 10,950 images were collected. From these images, 787,586 particles were picked, among which, 97,098 particles were finally used to construct the structural model at 2.68-Å resolution (figs. S2 and S3 and table S3) (see Materials and Methods for more details). The structure is a dimer and shows a unique organization, in which one monomer contains a PSII core, one FCPII dimer associated at each side of the PSII core, and three FCPII monomers (Fig. 2). Among the two FCPII dimers, one is a homodimer composed of Lhcf7 and associates with the PSII core through an Lhcx6_1 monomer. The other one is a heterodimer composed of Lhcf6 and Lhcf11, which binds a monomeric Lhcf5 and is connected to the core mainly through the Lhca2 monomer (Fig. 2). The FCPII heterodimer and Lhcf5 and Lhca2 monomers are lost in the second PSII-FCPII monomer during purification (Fig. 2A and fig. S2), resulting in an overall molecular weight of 1.1 MDa for the supercomplex. In addition to the protein subunits, 191 Chls (183 Chls *a* and 8 Chls *c*), 29 Fxs, 10 Ddxs, 2 Dtxs, 22 BCR, 2 pheophytins, 4 hemes, 2 plastoquinones, 2 nonheme irons, 2 bicarbonate ions, 4 bound chloride ions, 2 Mn_4_CaO_5_ clusters, and 69 lipids are assigned (Fig. 3 and table S4). The inorganic cofactors (nonheme irons, bicarbonates, chlorides, and Mn_4_CaO_5_ clusters) are the same as those found in cyanobacteria (*6*, *7*).

The PSII core contains 4 large, transmembrane subunits (D1, D2, CP47, and CP43), 14 small transmembrane subunits (PsbE, PsbF, PsbH, PsbI, PsbJ, PsbK, PsbL, PsbM, PsbT, PsbW, PsbX, PsbY, PsbZ, and Unknown1) (Fig. 2B and fig. S3A), and 5 extrinsic subunits (PsbO, PsbQ, PsbU, PsbV, and Psb31) attached at the lumenal surface for oxygen evolution (Fig. 2C). Most of these subunits are conserved from cyanobacteria, red algae, and the diatom *C. gracilis*. However, compared with the previous structure of Cg-PSII-FCPII (*24*, *25*, *36*), the subunit Cg-PsbG nearby CP47 is not found in the Tp-PSII-FCPII supercomplex (fig. S4A). The S-tetrameric FCP-A coupled with PsbG may therefore be unable to bind to Tp-PSII-FCPII, which is replaced by an Lhcf7 homodimer associated with Unknown1 and Lhcx6_1 (Fig. 2B). The Psb31 extrinsic subunit is retained, which enhances the oxygen-evolving activity in diatoms.

The unique organization of Tp-PSII-FCPII is largely different from the C_2_S_2_M_2_-type organization of PSII-FCPII isolated from another centric diatom *C. gracilis* (*24*, *25*, *36*), or the typical C_2_S_2_M_2_-type PSII-LHCII of higher plants (*10*), or the Tp-PSII-FCPII particles solved by EM (*34*) and PSII-FCPII in two diatom species solved by in situ cryo-ET (*34*, *37*), in that the major FCPIIs in Tp-PSII-FCPII are dimers in contrast to the tetrameric or trimeric FCPIIs or trimeric LHCIIs found in other organisms. The LHCX-type antenna Lhcx6_1 was found in the PSII supercomplex and located close to PsbX, PsbH, and D2 (Fig. 2). An Lhca2 subunit was found in the same location as Cg-FCP-D in the PSII-FCP-A supercomplex of *C. gracilis* (*24*, *25*, *36*). This Tp-Lhca2 has a similar structure with Cg-FCP-D (Cg-Lhcr17) (fig. S5) and may mediate the binding of the peripheral heterodimer to the PSII core with its special long C-terminal loop.

The FCPII homo- and heterodimers in *T. pseudonana* are connected by two helices C from two monomers (Fig. 3, A and B) similar to the isolated FCP dimer solved previously (*33*). The Lhcf7 homodimer (named Lhcf7_1 and Lhcf7_2, respectively) is connected to the CP47 side of the PSII core through Lhcx6_1 (fig. S6A). The Lhcf6 and Lhcf11 heterodimer is bound to the CP43 side of the PSII core weakly through Lhca2 (fig. S6, B and C). Moreover, the locations of the three FCPII monomers in Tp-PSII-FCPII are largely different from those of FCPII monomers in Cg-PSII-FCPII (*24*, *25*, *36*) and LHCII monomers found in green algae and higher plants (*10*–*12*). These differences may be caused by some differences in the small subunits of the PSII core between different organisms and illustrate the unique organization of the diatom PSII-FCPII supercomplex.

### Structure of the peripheral FCPIIs

According to the sequence alignment and phylogenetic analysis (fig. S1), Lhcf7 and Lhcf11 of *T. pseudonana* belong to a unique Lhcf branch different from Lhcf4 of *P. tricornutum* and Lhcf1 of *C. gracilis*. The sequence identity between Lhcf7 and Lhcf11 of *T. pseudonana* is high (60%), whereas identities between Tp-Lhcf7/Lhcf11 and Pt-Lhcf4/Cg-Lhcf1 are around 30% (table S5). Tp-Lhcf7 has similar secondary structures in the N-terminal loop, BC-loop, and three transmembrane helices with those of Pt-Lhcf4 and Cg-Lhcf1 (fig. S7A), whereas its AC-loop is shorter and the C-terminal helix D is absent, indicating that Tp-Lhcf7 could not assemble into tetramers as Cg-Lhcf1. Although the monomer-monomer interactions in the Tp-Lhcf7 homodimer is similar to those of the Pt-Lhcf4 homodimer, Tp-Lhcf7 has a longer C-terminal loop than Pt-Lhcf4, making it able to interact with Tp-Lhcx6_1 (figs. S1A and S7A).

**Fig. 4.**
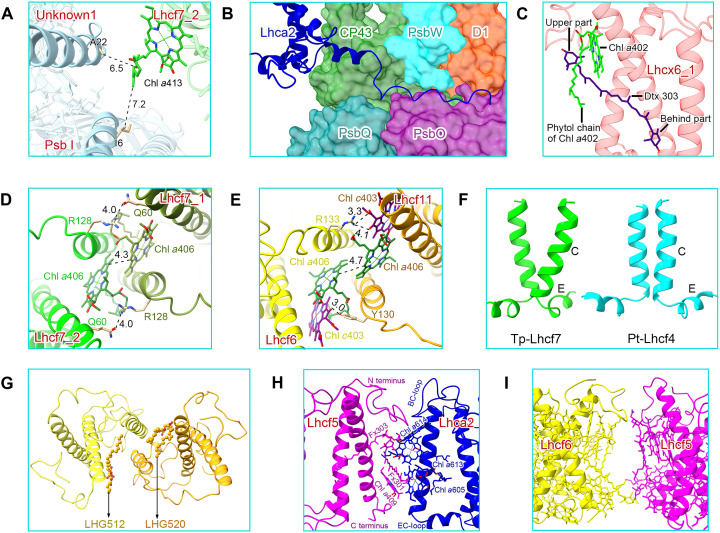
Characteristics of FCPIIs and some typical, important interactions among them. (**A**) Chl *a*413 of Tp-Lhcf7_2 weakly interacts with PSII core subunits Unknown1 and PsbI. (**B**) The long C terminus of Lhca2 extends to the PSII core and interacts with CP43, PsbW, D1, PsbQ, and PsbO at the luminal surface region. (**C**) The upper part of the Dtx molecule of Lhcx6_1 is embedded in the phytol chain of Chl *a*402. (**D**) Monomer-monomer interactions between the two C helices with a view from the stromal side in the Tp-Lhcf7 homodimer. (**E**) Monomer-monomer interactions between the two C helices of the Tp-Lhcf11-Lhcf6 heterodimer on the stromal side. (**F**) Comparison of the Tp-Lhcf7 (green) and Pt-Lhcf4 (cyan) homodimer structures. The former has a larger gap between two helices C than the latter in the stromal side; hence, their lumenal sides are closer to each other. (**G**) Two lipids are located in the interface of the Tp-Lhcf11-Lhcf6 heterodimer. (**H**) Interactions between Tp-Lhcf5 and Tp-Lhca2. (**I**) Interactions between Tp-Lhcf5 and Tp-Lhcf6.

Tp-Lhcf7_1 binds 12, and Lhcf7_2 binds 13 Chls (Fig. 3A), among which 9 (Chls 401 to 409) are conserved in Pt-Lhcf4 and Cg-Lhcf1 with respect to both positions and orientations (fig. S8, A and B). Compared with Cg-Lhcf1, Chl *a*410 of Tp-Lhcf7 is shifted to the stromal side and coordinated by its specific AC-loop, and the additional Chl 411/412/413 in Tp-Lhcf7 are located close to helices A/B and C terminus at the lumenal side, respectively (fig. S8, A and B). Chl *a*413 in Lhcf7_1 of the Tp-Lhcf7 homodimer is lost because of a loose binding, but this Chl *a*413 is strongly bound in the gap region between Lhcf7_2 and the PSII core and thus visible in the structure of *T. pseudonana* (Figs. 3A and 4A). All Chls in Tp-Lhcf7 are identified as Chls *a*; hence, the phytol chains of Chl *a*403 and *a*408 will conflict with other carotenoids in the protein scaffold, which leads to the loss of Fx306 and Fx307 in Tp-Lhcf7 that are otherwise found in Pt-Lhcf4 (fig. S8A). There are two Fxs, Fx301 and Fx302, in Tp-Lhcf7, which are similar to those found in the previous homodimeric (*33*) and tetrameric FCPs (*24*, *25*, *36*). Two central carotenoid sites embedded in the grooves formed by the crossing helices A and B in Pt-Lhcf4 were occupied by two Ddxs (Ddx303 and Ddx305) in Tp-Lhcf7 (Fig. 3A and fig. S8, A and B). Because of a different structure of the AC-loop, Fx304 is absent in Tp-Lhcf7. The loss of these Fxs and Chls *c* in Tp-Lhcf7 suggests its weak ability in harvesting blue-green light, and the presence of two Ddx molecules may suggest an important role in energy quenching.

Among the FCP heterodimer formed by Tp-Lhcf6 and Tp-Lhcf11 (Fig. 3B and fig. S3C), Tp-Lhcf11 is similar to Tp-Lhcf7 in both protein structure and pigments bound and only exhibits several small differences in the loop structures and two changes of pigment species at Chl *c*403 and Fx305 sites (Fig. 3, A and B, and figs. S3D and S7B). However, Tp-Lhcf6 is classified into the Pt-Lhcf4 group based on sequence alignment and phylogenic analysis (figs. S1 and S7C and table S5). Tp-Lhcf6 contains conserved Chls 401 to 409 and Fxs 301 to 307 as those in Pt-Lhcf4 and similar to Cg-Lhcf1 (Fig. 3B; fig. S8, C and D; and table S6), among which Chl *c*403/*c*408 and Fx 304/306/307 are responsible for blue-green light harvesting. An extra Chl *a*412 is found at its C-terminal loop which is extremely shorter than that of Pt-Lhcf4, thus Tp-Lhcf6 contained 10 Chls and 7 Fxs in total (Fig. 3B and table S6).

Tp-Lhcf5 has a high sequence identity with that of Pt-Lhcf4 (62%; see fig. S7D and table S5), and they bind nine identical Chls (Chls 401 to 409 including Chls *c*403/*c*408) and six identical Fxs (Fxs 301 to 307 excluding Fx304). The only additional Chl *a*410 in Tp-Lhcf5 is found in helix E (Fig. 3C). However, Tp-Lhcf5 is located at the peripheral of Lhca2 in a monomeric state but not in the typical dimeric assembly (Fig. 2B).

Other monomeric FCP subunits were identified as Tp-Lhca2 and Tp-Lhcx6_1 (figs. S3C and S4A), respectively, and they function as bridging antennas for the binding of FCP dimer to the PSII core. Tp-Lhca2 is the largest FCP subunit with a molecular weight of 24.8 kDa, which is analogous to Cg-FCP-D (Lhcr17) monomer in *C. gracilis* with 51% sequence identity (figs. S5A and S7F and table S5). Tp-Lhca2 and Cg-FCP-D were suggested as Lhca-like antennas of PSI (*22*), and they play bridging roles between the PSII core and the exterior FCPs analogous to CP29 in the PSII-LHCII supercomplex (*10*). The helices D and long C-terminal loops of both Tp-Lhca2 and Cg-FCP-D extend to the lumenal surface to enhance interactions with the PSII core subunits (Fig. 4B and fig. S6C). However, Tp-Lhca2 accommodates two extra Chls *a*613 and *a*614 by its twisted AC loop and loses the Fx617 site found in Cg-FCP-D (fig. S5C and table S7). The remaining 10 Chl sites and 2 carotenoid sites are very similar between Tp-Lhca2 and Cg-FCP-D, except for a small change of Chl *a*605 close to helix C (fig. S5C). Their different N-terminal loops suggest different roles in mediating interactions of various peripheral FCPs with the PSII core in different diatom species (figs. S4A and S7F).

**Fig. 5.**
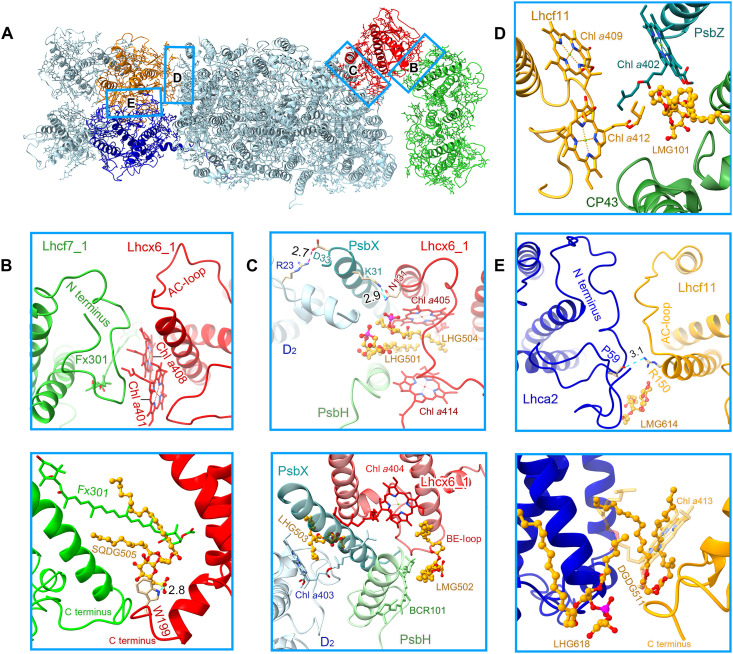
Typical, important interactions between FCPII and PSII core and among FCPIIs. (**A**) Overall structure of the PSII-FCPII supercomplex. The areas circled by rectangles correspond to the enlarged panels shown in the following, among which the stromal and lumenal side interactions are described in the top and bottom of (B), (C), and (E), respectively. (**B**) Interactions between Lhcx6_1 and Lhcf7_1, at the stromal and lumenal sides, respectively. (**C**) Tight interactions formed between Lhcx6_1, PsbX, PsbH, and D2. (**D**) Weak interactions between Lhcf11 and the PSII core. (**E**) Interactions between Lhca2 and Lhcf11. Lipid molecules are shown as ball and sticks, and their carbon atoms are colored orange, phosphorus atoms are colored magenta, and sulfur atoms are colored yellow. The dashed lines in cyan and purple indicate hydrogen and ionic bonds between adjacent subunits, respectively. Numbers indicate distances (angstrom).

Lhcx6_1 is an Lhcx family LHC subunit whose structure is solved by cryo-EM in the present study and has been proven to bind pigments (Fig. 3D). Lhcx6_1 contains three transmembrane helices (A, B, and C) and two short amphipathic helices (D and E) parallel to the lumenal surface, which is similar to the structures of other FCPs and LHCs (Fig. 3D and fig. S3C), despite a low sequence similarity with other Lhcf proteins (fig. S1 and table S5). Its helix B and helix C are longer than Lhcf subunits (figs. S1A and S7E), whereas the BE-loop is extremely short. Lhcx6_1 binds 11 Chls and 3 carotenoids (Fig. 3D and table S6); most of them are similar to other Lhcf subunits. An extra Chl *a*414 was found under the AC-loop of Tp-Lhcx6_1 (Fig. 3D and fig. S8, E and F), and the Car303 site of Tp-Lhcx6_1 (Lut621 site in LHCII) was identified as a diatoxanthin (Dtx303; fig. S3E). The upper part of the Dtx molecule extends to a position able to interact with the phytol chain of Chl *a*402 (Fig. 4C), whereas the lower part (including the ionon ring where the de-epoxidation takes place) is exposed to the periphery of the Lhcx6_1 subunit in the PSII supercomplex. Furthermore, there are five lipid molecules in the periphery of Lhcx6_1, which enhance its interactions with FCPs and the PSII core (Fig. 5, B and C). Among these lipid molecules, the head of SQDG505 connects to the C-terminal Trp199 of Lhcx6_1 by a hydrogen bond, and its fatty acid chain inserts into the space between Lhcx6_1 and Lhcf7_1, which may stabilize the binding of Lhcf7 homodimer in the supercomplex.

### Organization of FCPs and their interactions with the PSII core

Seven FCPII antennas associated with each PSII core of *T. pseudonana* have different organizations and interactions than those of FCPIIs found in *C. gracilis* (Fig. 5A and fig. S4A). In Tp-PSII-FCPII, both homodimeric and heterodimeric FCPIIs are assembled in a helix C–to–helix C manner similar to that of isolated Pt-Lhcf4 dimer (*33*), which is distinctly different from the helix A–to–helix C association of Cg-Lhcf1 tetramer and LHCII trimer of higher plants. A specific feature in both Tp-FCP homodimer and heterodimer is the formation of hydrophobic interactions by two helices C in conjunction with two Chls *a*406. Unlike the strong hydrogen bonds formed in AC-loop of the Pt-Lhcf4 dimer, interactions between Arg^128^ and Gln^60^ in the Tp-Lhcf7 homodimer, as well as between Chl *c*403 and Arg^133^ or Tyr^130^ in the Tp-Lhcf6-Lhcf11 heterodimer, are weaker on the stromal side (Fig. 4, D and E). Because the two helices C form strong hydrophobic interactions with adjacent helix E (Fig. 4F), monomers in the Tp-Lhcf7 homodimer and Tp-Lhcf11-Lhcf6 heterodimer are closer at the lumenal side. Consequently, Tp-Lhcf7 homodimer and Tp-Lhcf6-Lhcf11 heterodimer have a bigger gap between the two helices C at the stromal side than that of the two coupled helices C in Pt-Lhcf4 (Fig. 4F), enabling two Chls *a*406 to be located more closely, which may facilitate faster EET between the two monomers.

Tp-Lhcf7 has a longer C-terminal loop than Pt-Lhcf4, which may promote its interaction with Lhcx6_1 (Fig. 5B and fig. S7A). The C-terminal loop of one Lhcf7_1 in the homodimer forms hydrophobic interactions with Lhcx6_1 at the lumenal side, where SQDG505 is connected to Trp^199^ of Lhcx6_1 by a hydrogen bond. SQDG505 of Lhcx6_1 and Fx301 of Lhcf7_1 insert their two end groups into the gap space between Lhcf7_1 and Lhcx6_1, which may enhance their connections. At the stromal side, Tp-Lhcf7 uses its N-terminal loop to form hydrogen bonds and hydrophobic interactions with Lhcx6_1. In addition, Chl *a*401 and *a*408 of Lhcx6_1 may enhance their interactions with Tp-Lhcf7 (Fig. 5B).

Lhcx6_1 interacts with the PSII core mainly via D2, PsbH, and PsbX and bridges the Tp-Lhcf7 homodimer with the PSII core (Figs. 2 and 5C). Lhcx6_1 has a shorter BE-loop than that of other FCPs; hence, it could accommodate the N-terminal loops of PsbH and PsbX and interacts with them through LMG502, BCR101, and Chl *a*404 at the lumenal side. Lys^31^ of PsbX is hydrogen-bonded to Asn^131^ of Lhcx6_1 at the stromal side. PsbH interacts with the AC-loop of Lhcx6_1 via Chl *a*414 (Fig. 5C). In addition, four lipids 501 to 504 of Lhcx6_1 play important roles in stabilizing its binding at both stromal and lumenal sides (Fig. 5C). Furthermore, Chl *a*404 of Lhcx6_1 and Chl *a*403 of D2 insert their phytol chains into an open space to enhance their hydrophobic interactions, enhancing the stable binding of Lhcx6_1 to the PSII core (Fig. 5C). As the other Tp-Lhcf7 (Lhcf7_2) interacts with Unknown1/PsbI very weakly by the phytol chain of an extra Chl *a*413 (Fig. 4A and fig. S6A), the Tp-Lhcf7 homodimer associates with the PSII core almost exclusively through Lhcx6_1 (Figs. 2B and 5B). These differences between the Tp-Lhcf7 homodimer and FCPII S-tetramer in Cg-PSII-FCPII are partly ascribed to the different binding of the small Cg-PsbG subunit, as shown in fig. S4A.

On the CP43 side, FCPs are associated with PSII mainly through Lhca-like antennas in both *T. pseudonana* and *C. gracilis* PSII-FCPII (Fig. 2 and fig. S4A). Tp-Lhca2 has a similar C-terminal loop as that of Cg-FCP-D (fig. S7F), which extends to the lumenal surface of CP43, PsbO, and PsbW to form interactions among them (Fig. 4B and fig. S6C). Tp-Lhca2 is crucial for the binding of the Tp-Lhcf6-Lhcf11 heterodimer partly because of the absence of FCP-E (Cg-Lhcf4) of Cg-PSII-FCPII in Tp-PSII-FCPII (fig. S4A). The long N terminus of Tp-Lhca2 and the AC-loop of Tp-Lhcf11 form tight, hydrophobic interactions at the stromal surface (Fig. 5E). In addition, a pair of hydrogen bond between Arg^150^ of Tp-Lhcf11 and Pro^59^ of Tp-Lhca2 and a large number of pigments and lipids contribute to the stable binding between Tp-Lhca2 and Tp-Lhcf11 (Fig. 5E). In contrast, weak interactions between the C-terminal loop of Tp-Lhcf11 and CP43/PsbZ are formed by flexible phytol chains of Chls or lipids (Fig. 5D and fig. S6B). Owing to these weak associations between Tp-Lhcf11 and the PSII core in *T. pseudonana* (*24*, *25*, *36*), the Lhca-like FCPs play an important role in binding of peripheral antennas to the PSII core.

**Fig. 6.**
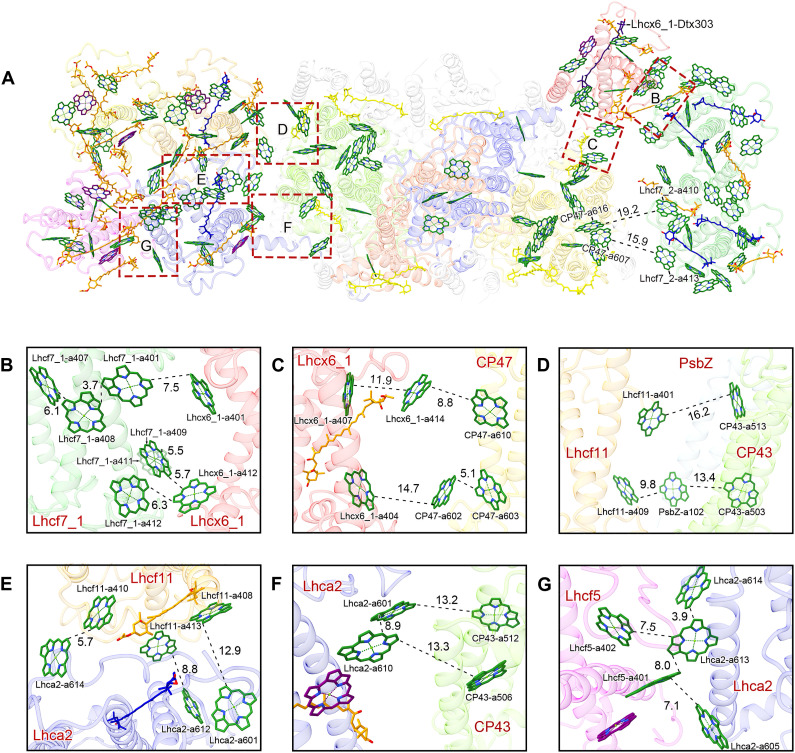
Pigment arrangement and possible EET pathways. (**A**) Pigment-pigment interactions in PSII-FCPII. The structure is viewed from the stromal side. Colors of the antenna subunits are the same as in Fig. 2. CP43, D1, D2, and CP47 are colored chartreuse, orange-red, blue, and gold, respectively. The pigments of Chl *a*, Chl *c*, Fx, and Ddx are colored forest green, magenta, orange, and blue, respectively, and shown as sticks. Only rings of the Chls are depicted. Red squared areas are enlarged in (B) to (G). Interactions are indicated by dashed lines, and numbers are distances in angstrom. (**B**) Pathways from Lhcf7_1 to Lhcx6_1. (**C**) Pathways between Lhcx6_1 and CP47. (**D**) Pathways between Lhcf11, CP43, and PsbZ. (**E**) Pathways between Lhcf11 and Lhca2. (**F**) Pathways between Lhca2 and CP43. (**G**) Pathways between Lhcf5 and Lhca2.

Tp-Lhcf6 in the heterodimer has a longer AC-loop and a shorter C terminus than Tp-Lhcf11 (Fig. 3B), which is unsuitable to interact with Tp-Lhca2. The interactions within the Tp-Lhcf6-Lhcf11 heterodimer are similar to those in the Tp-Lhcf7 homodimer (Fig. 4, D and E), such as interactions between the coupled helices C and Chls *a*406. Furthermore, LHG520 of Tp-Lhcf11 and LHG512 of Tp-Lhcf6 are inserted into the interface between the two monomers and strengthen their associations (Fig. 4G). Tp-Lhcf5 was associated at the outside of Tp-Lhca2 loosely by its N- and C-terminal loops (Fig. 4H) and has no direct interactions with Tp-Lhcf6 (Fig. 4I). Both Tp-Lhcf5 and Tp-Lhcf6 are in the periphery of the supercomplex and rich in Fx; thus, they may harvest more green light (Fig. 6A and table S6).

### Light harvesting and energy transfer from FCP antennas to PSII core

FCPs of diatoms exhibit great diversity in their protein structures, pigment compositions, and assembling states, which include FCP monomers, dimers, tetramers, oligomers, and even flexible peripheral trimer (*24*, *25*, *33*, *36*, *38*). While Chl-binding sites 401 to 409 are conserved in all of the FCPIIs whose structures have been solved (*33*) except Tp-Lhcx6_1 where site 405 is lost (Fig. 3D and fig. S8E), the Chl sites 410 to 414 are located mainly in the loop regions and bind differently in different FCPIIs. These Chl sites may play important roles in modulating EET. From previous Pt-Lhcf4 and Cg-Lhcf1 structures (*24*, *25*, *33*, *36*), Chl *c* prefers to occupy 403, 405, and 408 sites. As seen in table S6, there are more Chls *c* and Fxs in Pt-Lhcf4/Cg-Lhcf1 and Tp-Lhcf 5/6 but fewer in other Tp-FCPIIs.

Among the binding sites of carotenoids, sites 303 and 305 are conserved; they are located in the central regions similar to the binding sites of lutein in Lhcb proteins (*4*, *5*). Fx304, Fx306, and Fx307 may have red-shifted absorptions to harvest green light (*33*) and are found only in Pt-Lhcf4 and Tp-Lhcf 5/6 but absent in Tp-Lhcf 7/11 and Lhcx6_1. Because of the binding of more Chls *c* and red-shifted Fxs (fig. S8, C and D), Pt-Lhcf4 and Tp-Lhcf 5/6 are suggested to have a greater ability to harvest blue-green light (470 to 550 nm). These pigment compositions also suggest that Tp-Lhcf 7/11 and Lhcx6_1 may not be good in blue-green light harvesting but probably serve as good linkers analogous to Tp-Lhca2 and Cg-FCP-D to collect and deliver the excitation energy to the reaction centers (tables S5 and S6).

Compared with the previous Pt-Lhcf4 dimer, Tp-Lhcf7 has faster energy equilibrium within the homodimer because of the smaller distance between Chls *a*406 at 4.3 Å (Fig. 4D) and strongly coupled Chls *a*402/*a*403/*a*406 and *a*401/*a*407/*a*408 clusters, which suggests that these Chls may provide the lowest energy level among the coupled Chls (Fig. 3A). The EET from Tp-Lhcf7 to Lhcx6_1 should be efficient by pathways at both stromal and lumenal sides. At the stromal side, the Chls *a*401/*a*407/*a*408 cluster of Tp-Lhcf7 may transfer energy to Chl *a*401 of Lhcx6_1 at a distance of 7.5 Å (Fig. 6B), whereas at the lumenal side, Chls *a*409 and *a*412 of Tp-Lhcf7 can transfer excitation energy to Chl *a*412 of Lhcx6_1 efficiently at distances of 5.7 and 6.3 Å (Fig. 6B), respectively. Lhcx6_1 uses two pathways to transfer energy collected by the Tp-Lhcf7 homodimer to the PSII core, namely, from Chl *a*404 of Lhcx6_1 to Chl *a*602 of CP47 at 14.7 Å and from Chl *a*414 of Lhcx6_1 to Chl *a*610 of CP47 at 8.8 Å, respectively (Fig. 6C). Compared with the pathways via Lhcx6_1, EET from Lhcf7_2 to CP47 directly may be slower due to their large distance (Figs. 6A and fig. S9). Chls *a*410 and *a*413 of Lhcf7_2 could transfer energy to Chls *a*616 and *a*607 of CP47 at the stromal and lumenal side, respectively (Figs. 6A and fig. S9). The Tp-Lhcf7 homodimer may provide a faster EET because of the presence of additional Chls *a*410-*a*413 and the absence of Chl *c*, which may serve as a substantial relay for EET compared to other loosely associated FCPs (Fig. 3A). However, most EET pathways of the Lhcf7 homodimer rely on Lhcx6_1 to complete EET to the PSII core (fig. S9).

In the Tp-Lhcf6-Lhcf11 heterodimer, there are three Chls *c* among EET pathways (Fig. 3B), which may lead to slower equilibration of energy between Tp-Lhcf6 and Tp-Lhcf11. The Chls *a*401/*a*407/*a*408 cluster in Tp-Lhcf11 may serve as an important exit of EET. However, the EET pathway from Chls *a*401 of Tp-Lhcf11 directly to CP43 may be less efficient than that from Chl *a*408 of Tp-Lhcf11 to Chl *a*601 of Tp-Lhca2 at the stromal side, due to a longer distance of the former (Fig. 6, D and E). At the lumenal side, direct EET from Tp-Lhcf11 to the PSII core relies on Chl *a*409, which builds a fast pathway via Chl *a*102 of PsbZ (9.8 Å) and then to Chl *a*503 of CP43 (13.4 Å) (Fig. 6D). Owing to tight interactions between Tp-Lhcf11 and Tp-Lhca2, more efficient EET pathways are formed by Chl *a*410 of Tp-Lhcf11 to Chl *a*614 of Tp-Lhca2 (5.7 Å) and Chl *a*413 of Tp-Lhcf11 to Chl a612 of Tp-Lhca2 (8.8 Å) (Fig. 6E). Tp-Lhca2 may transfer excitation energy via Chl *a*601/*a*610 to CP43 (Fig. 6F), which is the same as seen in Cg-FCP-D. As a result, the Tp-Lhcf6-Lhcf11 heterodimer has multiple pathways to transfer excitation energy to the PSII core.

A monomeric Tp-Lhcf5 is rich in Fxs and forms “helix A–to–helix C” interactions with Tp-Lhca2 (Fig. 4H and table S6). The shifted Chl *a*605 and extra Chl *a*613 of Tp-Lhca2 may receive energy from Chl *a*401 and Chl *a*402 of Tp-Lhcf5 at distances of around 8.0 Å (Fig. 6G); thus, energy captured by Lhcf5 is eventually transferred through Lhca2 to the PSII core.

### Possible energy quenching associated with Lhcx6_1 and other FCPs

Diatoms need to cope with light-intensity fluctuations at a high frequency (*39*). Non-photochemical quenching (NPQ) is trigged under high light conditions to protect the photosystems, under which photochemical reactions are enhanced, causing accumulation of protons (*40*). Diatoms can adapt to light fluctuations rapidly by changing their antenna status between efficient light harvesting and super NPQ, the latter is closely associated with the LI818 clade LHCX and epoxidation/de-epoxidation cycle of Ddx/Dtx (*20*, *41*). Lhcx6_1 is encoded by an *lhcx* gene and has been shown to exist in two closely related centric diatom species *C. meneghiniana* and *T. pseudonana* (*21*). In some typical central diatoms, NPQ is suggested to involve two major pathways, namely, a transient light-induced qE1 pathway coupled with light-driven ΔpH and a continuous qE2 pathway relying on the Ddx-Dtx cycle (*40*–*42*). Our HPLC analysis demonstrated the increase of Dtx in cells grown under high light (Fig. 1F), which is the same as those reported recently (*43*). Thus, the Ddx/Dtx cycle and Dtx-dependent NPQ component may respond to stress in the Tp-PSII-FCPII supercomplex under high-light conditions. As shown in Fig. 2 (A and B), Lhcx6_1 is located in a position between the Tp-Lhcf7 homodimer and PSII core, and Dtx303 in Lhcx6_1 is located in close proximity with the Chl *a*402-*c*403-*a*406 clusters but is deviated from the direct and efficient pathway for EET via its Chl *a*401-*a*407-*a*408 cluster (Figs. 3D and 6A). Therefore, we speculate that this Dtx may serve as an efficient energy-quenching site in Lhcx6_1. Lhcx6_1 may thus be involved in the proposed qE1 photoprotective pathway to avoid photodamage to the PSII core. This agrees with the previous biochemical and spectral analysis that Lhcx6_1 is a good candidate to be involved in the proposed qE1 photoprotective pathway, which relies on a quenching site close to the core, and the formation of a quenching site close to the core is dependent on Dtx (*21*, *40*, *42*, *43*). Other Lhcx proteins may mediate the aggregation and quenching of more flexible peripheral FCPs.

## DISCUSSION

The peripheral antennas of PSII are important for light harvesting and adaptation to various light conditions. Great differences are found in the organizations of LHCII and FCPII antennas and the arrangement of pigments among them, between the PSII-LHCII supercomplexes of green algae, vascular plants, and PSII-FCPII of diatoms such as *C. gracilis* (*33*, *44*) and *T. pseudonana* (*34*). FCPs showed a greater diversity in their organizations ranging from monomer, dimer, trimer, tetramer, and oligomer (*24*, *25*, *33*–*36*, *38*) in comparison with LHCII of green algae and higher plants that mainly form trimer and monomer (*10*–*13*). In addition, a member of Lhcx family Lhcx6_1 is associated with PSII (*21*, *45*), which is possibly involved in energy quenching. This indicates that diatoms have strong abilities to deal with various light environments, which may be an important factor for the success of diatoms as dominant photosynthetic species in the oceans.

In this study, we solved the structure of PSII-FCPII binding dimeric FCP antennas and a member of the Lhcx family Lhcx6_1. Dimeric FCP has been found in isolated FCPs previously (*33*) but was found to be associated with PSII from *T. pseudonana* in the present study. Two types of dimers are present, one is a homodimer formed by Lhcf7 and associated with the CP47 side through Lhcx6_1, whereas another one is a heterodimer formed by Lhcf6 and Lhcf11 and associated with the CP43 side through interactions with another FCPII monomer, Lhca2 (fig. S10, A and B). Previous studies showed that the strongly associated (S) and moderately associated (M) tetramers in the diatom *C. gracilis* are opposite to S/M trimers of green-lineage photosynthetic organisms, where S/M trimers are located at the CP43 and CP47 sides, respectively (fig. S10C) (*10*–*12*). This difference may be caused by the differences in the small trans-membrane subunits present in different PSII cores. In diatoms, PsbX and the extra Unknown1 participate in binding the homodimer directly or indirectly in *T. pseudonana*, and the unique PsbG promotes the binding of S-tetramer in *C. gracilis* (*24*, *25*, *36*), whereas in higher plants, CP29 play a bridging role between the M-trimer and the PSII core (*10*). On the other hand, diatoms use an Lhca-like protein, Tp-Lhca2 or Cg-FCP-D, to connect heterodimer or M-tetramer to PsbW of the PSII core (*24*, *25*, *36*), in analogy to the role of CP29 in higher plants, whereas the association of the FCP homodimer is mediated by an Lhcx family antenna Lhcx6_1 protein.

In a previous study (*34*), the structure of Tp-PSII-FCPII showed a C_2_S_2_-type organization, in which FCPII are organized into two strongly associated trimers, by EM and cryo-ET at 19-Å resolution. This organization is different from the results resolved at 2.68 Å in this study. We compared the biochemical studies of the authors group (*21*, *34*, *43*, *45*) with the present study and found that our samples are very similar to those of the previous one based on mass spectrometry and HPLC results (*43*, *45*). A small difference is found in the fluorescence emission spectra at 77 K in that our sample had a shorter emission wavelength than the previous study (*43*). This may be caused by partial loss of FCPIIs in the second monomer in our sample, because we removed 1 M betaine by gel filtration chromatography in the last step of purification (see Materials and Methods) immediately before cryo-EM sample preparation, to obtain a high-resolution by cryo-EM. The removal of betaine may damage the supercomplex and cause the release of the loosely connected FCPs from the complex, resulting in a shorter wavelength of fluorescence emission at 77 K. However, this will unlikely change the oligomerization state of the FCPs, and the trimeric organization of FCPIIs may be due to the low resolution achieved in the previous study (*34*). We cannot exclude the presence of FCP trimers in *T. pseudonana*; however, they are not associated with PSII immediately and may therefore associate with PSII at the outer periphery.

The dimeric organization of FCPIIs found in *T. pseudonana* PSII-FCPII is different from the tetrameric organization found in PSII-FCPII of another diatom *C. gracilis* (*24*, *25*, *36*). Phylogenetic tree analysis (fig. S1B) showed that Tp-Lhcf7/11 forming the dimer and Cg-Lhcf1/5/6/7 forming the tetramer are divided into two obvious branches. The FCPII tetramers have greater advantages in light harvesting, as their carotenoids are all Fxs which are more efficient in blue-green light harvesting. On the other hand, the dimeric FCPII dimers are rich in Ddx, which may impose a role in energy quenching (fig. S4C). The difference in the binding strength of FCPII tetramers and dimers in PSII-FCPCII of *C. gracilis* and *T. pseudonana* may be due to the presence of PsbG in the former (*24*, *25*, *36*) but not in the latter. We are not sure whether PsbG is also present in the *T. pseudonana* genome, as the sequence of PsbG is not known. No Tp-Lhcf proteins are found to be similar to Cg-Lhcf1 homologs (fig. S1B); therefore, the presence or absence of PsbG and the different associations of FCPII dimers or tetramers in different diatoms may be ascribed to evolutionary adaptations to the different environments that each diatom occupies. The structural heterogeneity in the PSII supercomplex may therefore assist different diatoms in adapting to different light conditions.

In *T. pseudonana*, the Lhcx family antenna Lhcx6_1 binds a number of Chls and carotenoids. The only Dtx molecule found in the PSII-FCPII supercomplex is also present in this Lhcx subunit. This may suggest a role of this subunit in energy quenching. The specific Dtx303 is bound at the edge of the protein subunit with the help of phytol chain of Chl *a*402 in Lhcx6_1 (Figs. 4C and 6A). If this Dtx site is affected by the enzymatic reaction of the Ddx/Dtx cycle, then the lower part (including the ionon ring where the de-epoxidation takes place) in Dtx303 near the edge of the supercomplex has more contact space for the access of enzymes than other pigments embedded in the supercomplex (Fig. 4C). On the other hand, the Ddx-rich Lhcf7 homodimer may also have a function in energy quenching, and the relatively loosely bound FCP heterodimer may easily detach from the PSII core, which could make the PSII core avoid the photodamage under high-light conditions. This may be one of the reasons why the heterodimer as well as Lhcf5 and Lhca2 are lost in one side of the PSII-FCPII dimer.

In conclusion, the cryo-EM structure of PSII-FCPII from *T. pseudonana* solved in this study showed detailed structural features of FCP dimers and the association of a member of the Lhcx family subunit, as well as a unique assembly pattern of PSII-FCPII different from PSII-FCPII of *C. gracilis* and PSII-LHCII of green algae and higher plants. Lhcx6_1 is associated with the PSII core, and its possible photoprotective role is proposed based on its location in the supercomplex and the binding of Dtx, which provides a structural basis for elucidating the mechanism of NPQ in diatoms. Different diatoms adapt to different light conditions by adopting different structural heterogeneity in the PSII supercomplex, which may be an important factor for the great survival success of this important group of red lineage species in the oceans.

## MATERIALS AND METHODS

### Purification and characterization of PSII-FCPII

*T. pseudonana* cells (GY-H27, Guangyu Biological Technology Co. Ltd. Shanghai, China) are cultured in the F/2 medium (*46*) by bubbling with 3% CO_2_-containing air, in a light intensity of 45 to 50 μmol photons m^2^ s^−1^ at a light/dark cycle of 16/8 hours under stirring at 135 rpm. The incubation temperature is 22°C. When the culture reaches 5 × 10^6^ cells/liter, cells were collected by centrifugation at 6000*g* and resuspended in an ice-cold buffer of 50 mM 2-morpholinoethanesulfonic acid (Mes)–NaOH (pH 6.5), 10 mM MgCl_2_, 1 M betaine, 5 mM CaCl_2_.

PSII-FCPII supercomplex was isolated with the procedure reported previously (*24*) at 4°C. Briefly, cells were disrupted by French Press (3 cycles at 30 MPa), and the unbroken cells were removed by centrifugation at 1000*g* for 15 min. Thylakoid membranes were collected by centrifugation at 40,000*g* for 30 min at 4°C and resuspended in a buffer of 50 mM Mes-NaOH (pH 6.5), 1 M betaine, 10 mM NaCl, 5 mM CaCl_2_ (MBNC). The thylakoid membranes were subsequently solubilized with 0.75% (w/v) *n*-dodecyl-α-d-maltopyranoside (α-DDM) (Anatrace, Maumee, OH) for 30 min on ice and centrifuged at 40,000*g* for 15 min to remove unsolubilized materials. The supernatant was loaded onto a linear sucrose density gradient (0.2 to 1.3 M sucrose) in the buffer MBNC containing 0.03% α-DDM and centrifuged at 248,000*g* for 18 hours.

After centrifugation, several major bands were obtained. In the upper side, the brown band is released FCPs, and the lower major bands are PSI-FCPI and PSII-FCPII. The lower down bands in the sucrose density gradient are PSI/PSII aggregates, which are not suitable for structural analysis. The PSII-FCPII was collected and its sucrose and betaine were removed by gel filtration chromatography (Cytiva, Superose 6 Increase 10/300 GL) in a buffer of 50 mM Mes-NaOH (pH 6.5), 10 mM NaCl, and 5 mM CaCl_2_ (MNC) supplemented with 0.03% α-DDM. The peak eluted was collected and concentrated by an ultrafiltration centrifuge tube (molecular weight cutoff: 100 kDa; AMICON, Merck Millipore) to a concentration of 2 mg Chl/ml. All procedures were performed under dim green light at 4°C or on ice.

### Characterization of PSII-FCPII

Subunit compositions of thylakoid membranes and purified PSII-FCPII supercomplexes from *T. pseudonana* were analyzed by SDS-PAGE using a gel containing 14% polyacrylamide and 7.5 M urea. The gel was stained by Coomassie brilliant blue R-250 (Sigma-Aldrich).

The identities of proteins in the gel were identified by mass spectrometry. The stained bands were cut out by SmartSlicer (AstroPrint), and the proteins in the gel were digested and extracted using sequencing-grade modified trypsin, followed by mass spectrometric analysis.

Room temperature absorption spectra were measured with an ultraviolet-vis spectrophotometer (Shimadzu, Japan) in the MNC buffer containing 0.03% α-DDM. Fluorescence emission and excitation spectra were measured at 77 K using a fluorescence spectrophotometer (F-7000, Hitachi, Japan) at a Chl concentration of 40 μg Chl/ml in the MNC buffer supplemented with 40% glycerol and 0.03% α-DDM.

Oxygen-evolving activity was measured by a Hansatech Clark oxygen electrode under saturating white light at 25°C in the buffer containing 50 mM Mes-NaOH (pH 6.5), 0.4 M sucrose, and 5 mM CaCl_2_ with the Tp-PSII-FCPII at 10 μg Chl/ml. Phenyl-*p*-benzoquinone (0.5 mM) was used as the electron acceptor. The oxygen-evolving activity of the Tp-PSII-FCPII was determined to be ~1240 mmol O_2_ (mg Chl)^−1^ hour^−1^.

### Pigment analysis

Pigment compositions of the PSII-FCPII supercomplex from *T. pseudonana* were analyzed by HPLC as described previously (*24*, *26*). Briefly, the samples collected from the sucrose density gradient were placed into 90% (v/v) acetone at 4°C to extract the pigments (*43*). After centrifugation at 14,400*g* for 10 min, the supernatant was injected into a C-18 reversed-phase column (5 μm, 100 Å, 250 mm by 4.6 mm, Grace, USA), and the pigments were eluted at 20°C at a flow rate of 1 ml/min with a linear gradient of buffer A (acetonitrile: water = 90:10) from 100 to 0% and buffer B (ethyl acetate) from 0 to 100%. Pigments were identified on the basis of their absorption spectra and elution times. The HPLC results indicated that the PSII-FCPII supercomplex contained Chl *a*, Chl *c*, Fx, Ddx, Dtx, and BCR.

To explore the changes in the pigment composition after light-stress treatment, *T. pseudonana* cells were cultured from 1 × 10^6^ cells/liter to 5 × 10^6^ cells/liter under light intensities of 150 and 50 μmol photons m^2^ s^−1^ as high light and low light, respectively. Then, the algal cells were collected, the PSII-FCPII supercomplex was purified as described above, and their pigment composition was analyzed.

### Cryo-EM image processing

The PSII-FCPII supercomplex purified from cells grown under low light was applied to a glow-discharged holey carbon grid (Quantifoil Au R2/1, 300 mesh) at a Chl concentration of 2 mg/ml and subsequently vitrified using a Vitrobot. The chamber of Vitrobot was set to 4°C and 100% humidity. The sample was blotted for 3 s with a blot force of 1. Cryo-EM images were collected with a Titan Krios microscope operated at 300 kV equipped with a Gatan Quantum energy filter, at a slit width of 20 eV, a spherical aberration corrector, a K3 camera (Gatan) operated at a superresolution mode, with ×81,000 magnification. Each movie comprises 32 frames with a total dose of ~60 e/Å^−2^ and a dose rate of 39 e^−^/pixel per s. Data acquisition was performed using the EPU software (Thermo Fisher Scientific) with a defocus range of −1.0 to −2.0 μm. The final images were binned, which results in a pixel size of 0.88 Å for further data processing.

A total of 10,950 movies are collected and processed with cryoSPARC (*47*), resulting in 787,586 particles which were boxed using crYOLO (*48*). Particles were extracted from micrographs, and two-dimensional (2D) classification was performed to discard bad particles. After several rounds of 2D classification, 538,802 particles were selected for 3D classification in cryoSPARC (*47*). Then, 97,098 particles were selected for nonuniform refinement using *C*1 symmetry, resulting in a density map with an overall resolution of 2.68 Å, estimated by the gold-standard Fourier shell correlation coefficient of 0.143. The local resolutions were estimated using cryoSPARC. To refine the peripheral FCPII, the local map was subtracted from the final full map (97,098 particles). The peripheral FCPII was reconstructed at 3.19-Å resolution based on the gold-standard FSC with a cutoff value of 0.143 using local refinement in cryoSPARC (*47*).

### Model building and refinement

Model building was carried out by applying the homology modeling method. The structure of *C. gracilis* PSII core [Protein Data Bank (PDB) code: 6JlU] and FCP subunits (PDB code: 6A2W) were placed and fitted into the 2.68-Å cryo-EM map with UCSF Chimera (*49*). On the basis of the results of mass spectrometric analyses, some sequences of the antenna and core subunits from *T. pseudonana* were built by SWISS-MODEL (*50*), and these subunits were subsequently checked manually and identified with COOT (*51*) in the 2.68- and 3.19-Å cryo-EM maps.

In the process of amino acid model building, we combined the biochemical mass spectrometry data (Fig. 1B) and biophysical cryo-EM density map data (fig. S2, B and C). On the basis of the density maps of amino acid residues in the 2.68 Å map, Lhcx6_1 and Lhcf7 were assigned (fig. S3C). Subsequently, with the help of the local density map at 3.19-Å resolution, Lhca2 was assigned on the basis of its longer C terminus, which allowed its stable binding to the PSII core and exhibited a good density map (fig. S3C). The heterodimer of Lhcf6 and Lhcf11 was identified by further analysis in the local resolution map, as Lhcf6 has a different AC loop compared to Lhcf11 (figs. S3C and S6, B and C). The last antenna subunit Lhcf5 is located at the edge area of the overall structure and shows a weaker density map. This subunit was assigned on the basis of the better density maps in the helices A and B regions (fig. S3C). We did not find any phosphorylation site in Thr/Ser/Tyr amino acid residues of the FCP proteins in PSII-FCPII by checking the density map at 2.68-Å resolution.

In the process of pigment model building, we combined the biochemical results of the HPLC analysis for PSII-LL (Fig. 1F) and biophysical cryo-EM density maps (fig. S2, B and C). We assigned Chl *a* or Chl *c* based on the difference in the density map at the phytol chain region (fig. S3B). In addition, the unique polar C-17 propionic acids of Chl *c* usually have interactions with the surrounding alkaline amino acids or Fx (table S8) (*33*). We were unable to distinguish between Chl *c*1 and Chl *c*2, because of their small differences and the resolution limit. Therefore, we assign either Chl *c*1 or Chl *c*2 based on the conserved Chl 408 (*c*1) or 403 (*c*2) site in the homologous 1.8-Å resolution structure of Pt-Lhcf4 (PDB: 6A2W). In the area where local resolution is less, Chl *c* in Lhcf11 is less certain. On the basis of the lack of density for the phytol chain and the same Chl *c* site compared with the homologous Pt-Lhcf4 (PDB: 6A2W), we assigned Chl *c*2 in Lhcf11 (fig. S4). For Chls *a*, most of the phytol chains can be modeled well, but in some Chls *a*, the local resolution in the edge areas is less clear. We therefore deleted some C atoms from the end of the phytol chain where the density maps for these phytol chains are less clear or not visible, to make all the chains match with the density map.

Regarding model building of carotenoids, Fx and Ddx were distinguished on the basis of the density covering the head group of carotenoids shown with a threshold of 0.14 contour level (step 1) by Volume Viewer in Chimera X (fig. S3E), as Fxs have more areas for the front four atoms (fig. S3E). In the area where local resolution is less, the Fx302 and Fx307 sites in Lhcf5 are less certain. On the basis of the same Fx sites in the homologous Pt-Lhcf4 structure (PDB: 6A2W), we preliminary assigned Fx for them. We assign Ddx/Dtx based on the thickness difference caused by the bulge of O atom (epoxy group) in Ddx (which makes the thickness of the lower ionon ring the same on both sides) (fig. S3E). The two molecules are very similar, and we determined from HPLC that Dtx is present. All the density maps for Ddx/Dtx molecules were checked, and Dtx303 in Lhcx6_1 most probably fits with the density map obtained in this study.

Last, the PSII-FCPII model was refined using Phenix (*52*), and the statistics for data collection and structure refinement are summarized in table S3. The structures in this paper were displayed with UCSF ChimeraX (*53*).

### Van der Waals force analyses within the adjacent subunits

The Contact function [default parameters: van der Waals (VDW) overlap ≥ −0.40A; only select “include intramodel”] of the ChimeraX software program was used to analyze VDW force interactions (*53*). The adjacent subunits used to analyze the interactions were chosen separately, the VDW forces inside the subunits were hidden respectively, and only the VDW forces formed by the adjacent subunits were shown (fig. S6).
