# Supplementary material for: Structure of a diatom photosystem II supercomplex containing a member of Lhcx family and dimeric FCPII
Source: Sci Adv. 2023 Oct 25;9(43):eadi8446. doi: 10.1126/sciadv.adi8446 (PMC10599620; doi:10.1126/sciadv.adi8446)
Supplement: Supplementary file 1 — Figs. S1 to S10 Tables S1 to S8 Legends for source data 1 to 8 [file sciadv.adi8446_sm.pdf]

Science Advances
AAAS

Supplementary Materials for

Structure of a diatom photosystem II supercomplex containing a member of Lhcx family and dimeric FCPII

Yue Feng *et al.*

Corresponding author: Wenda Wang, wdwang@ibcas.ac.cn; Jian-Ren Shen, shen@cc.okayama-u.ac.jp

Sci. Adv. **9**, eadi8446 (2023)
DOI: 10.1126/sciadv.adi8446

This PDF file includes:

Figs. S1 to S10
Tables S1 to S8
Legends for source data 1 to 8

Other Supplementary Material for this manuscript includes the following:

Source data 1 to 8

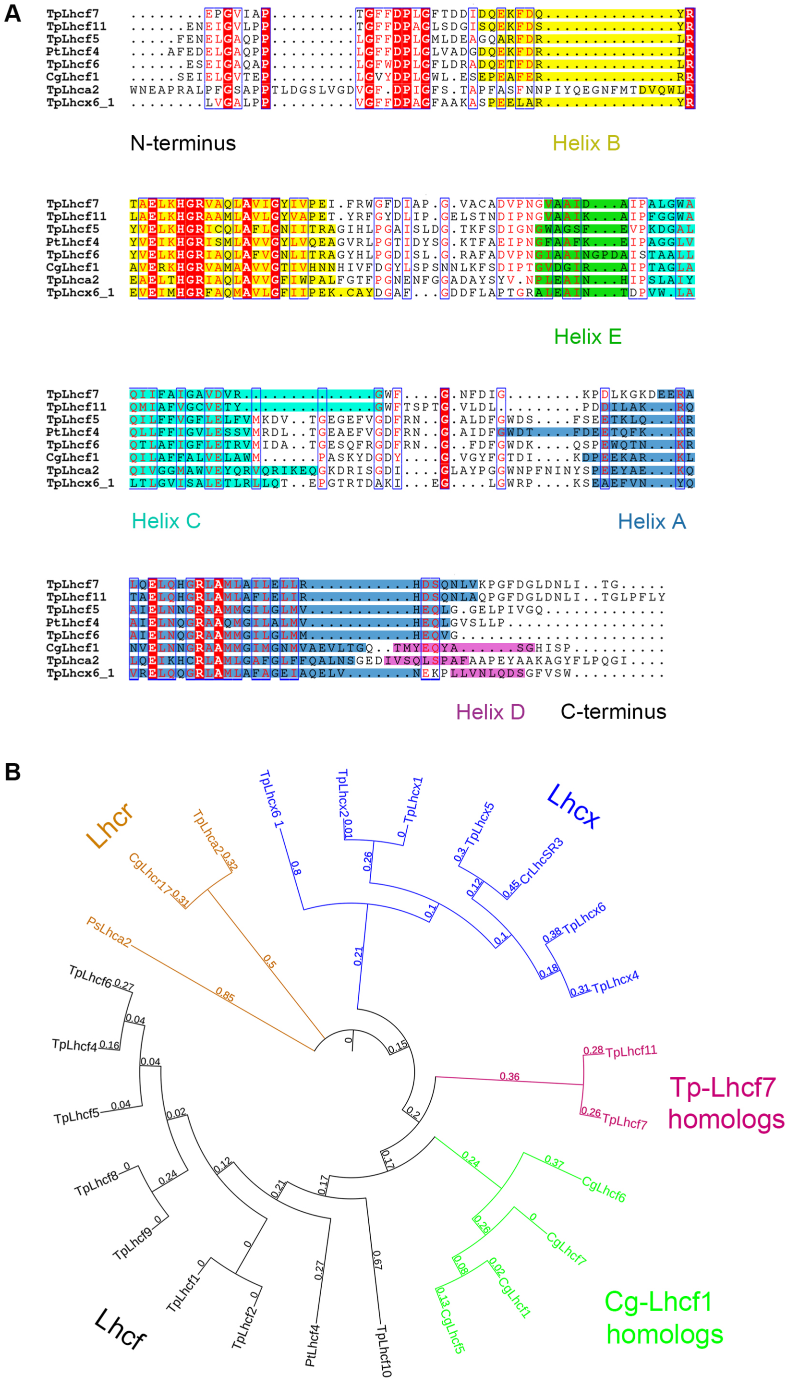

Fig. S1. Alignment of FCPII sequences and phylogenetic analysis.

(**A**) Sequence alignment of typical FCPIIs from three species of diatoms. Five helices in the secondary structure were colored differently. (**B)** Phylogenetic tree including all Lhcfs, Lhcxs and an Lhca2 (some of which are FCPIIs solved in this study) from *T. pseudonana* and some typical FCPIIs from *C. gracilis*, the Lhcf4 of *P. tricornutum* formed in FCP dimer, typical Lhca2 of *P. sativum* from higher plants, and photoprotective LhcSR3 of *C. reinhardtii*. The colors of clades are as follows: magenta, Tp-Lhcf7 homologs; green, Cg-Lhcf1 homologs that form FCP tetramer; orange, Lhca/Lhcr homologs; black, typical Lhcf antennas; blue, Lhcx homologs.

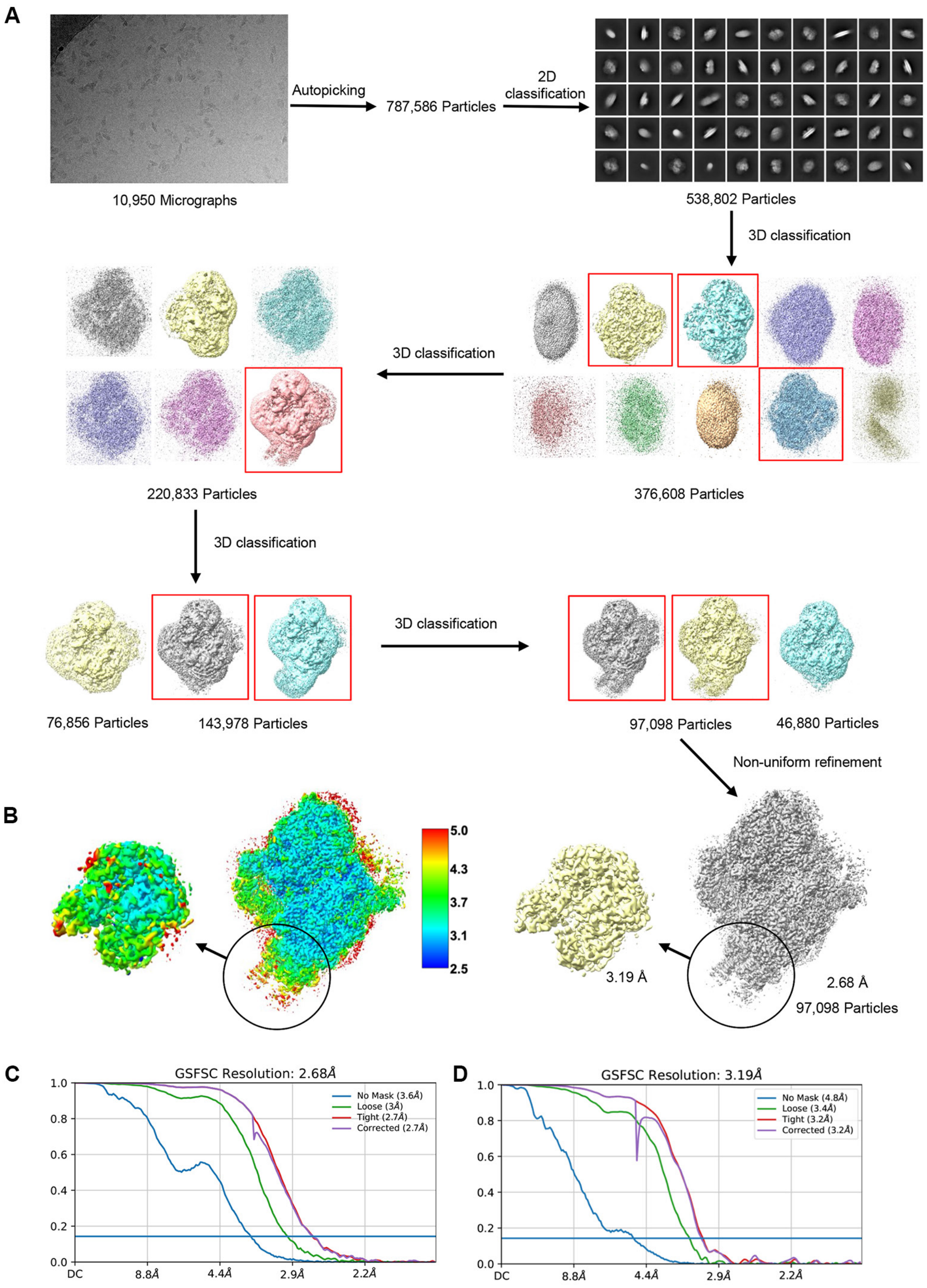

Fig. S2. Single particle cryo-EM analysis of the Tp-PSII-FCPII.

(A) A typical image of Tp-PSII-FCPII and flow chart of the cryo-EM data process. The global and local resolution maps of the PSII-FCPII were generated by cryoSPARC, in which four FCPs (Lhca2, Lhcf5, Lhcf11, and Lhcf6) were cycled and treated in a local map shown in panel **B**. **(B)** Resolutions (Å) of the global and local maps colored by cryoSPARC. (**C**) and (**D**) The gold standard FSC curves of the final 3D reconstruction for the global and local maps, respectively.

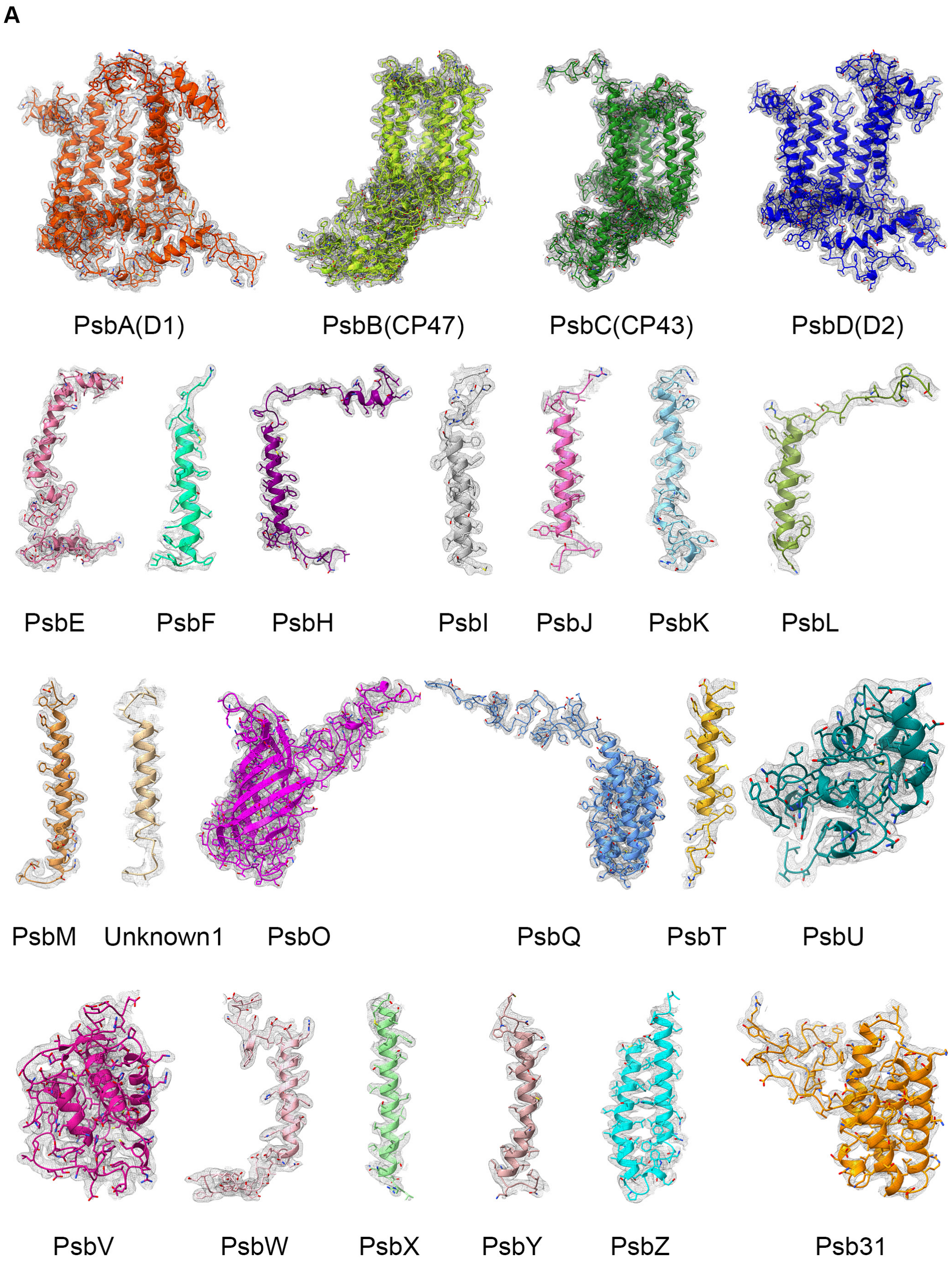
A
PsbA(D1)
PsbB(CP47)
PsbC(CP43)
PsbD(D2)
PsbE
PsbF
PsbH
PsbI
PsbJ
PsbK
PsbL
PsbM
Unknown1
PsbO
PsbQ
PsbT
PsbU
PsbV
PsbW
PsbX
PsbY
PsbZ
Psb31

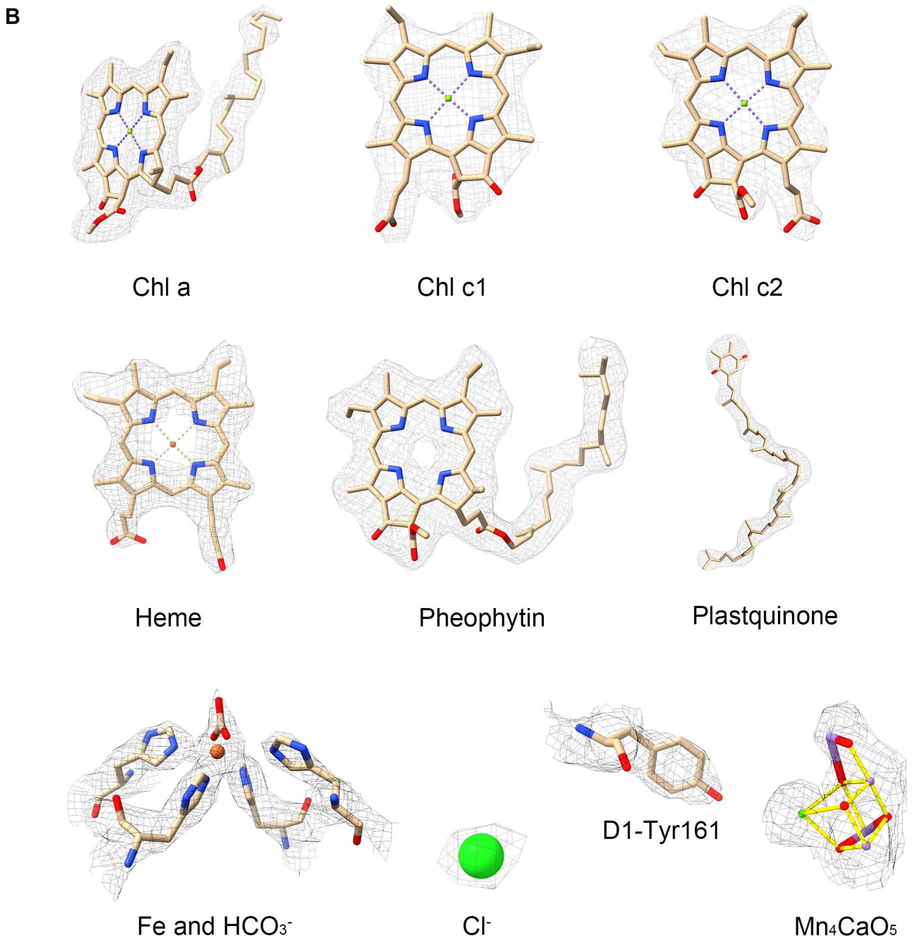
B
Chl a
Chl c1
Chl c2
Heme
Pheophytin
Plastquinone
Fe and HCO_3^-
Cl^-
D1-Tyr161
Mn_4CaO_5

c

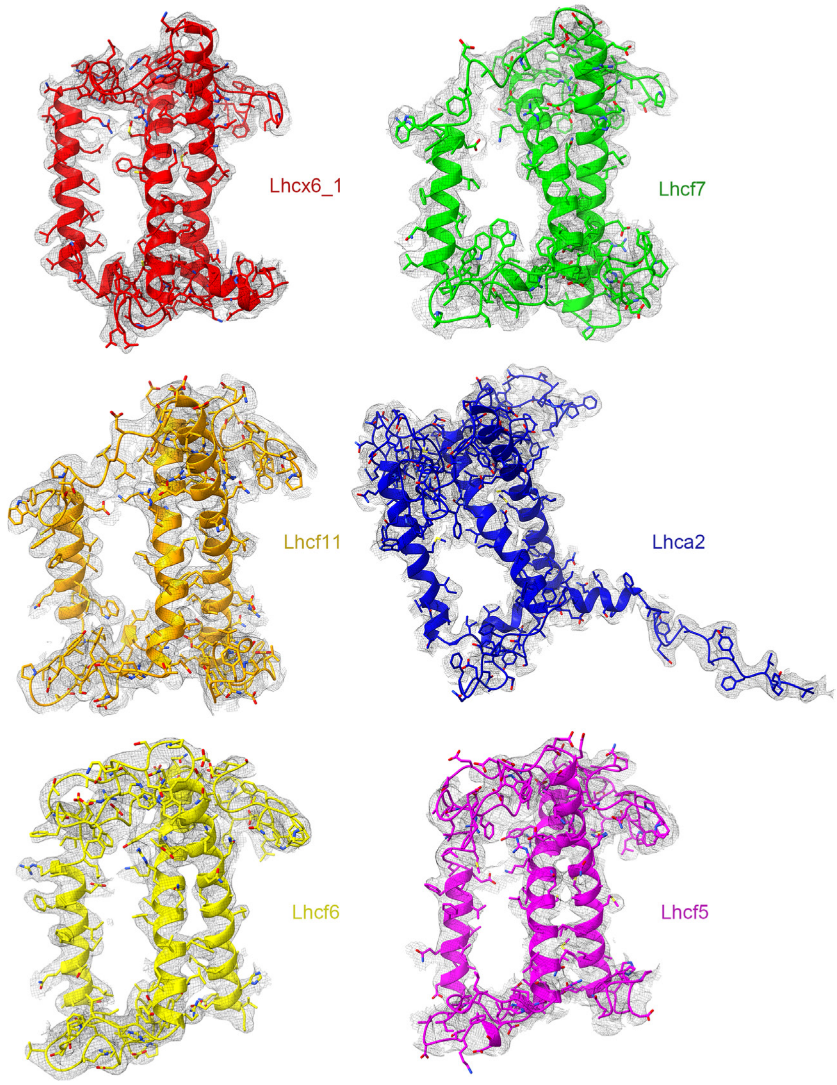

D

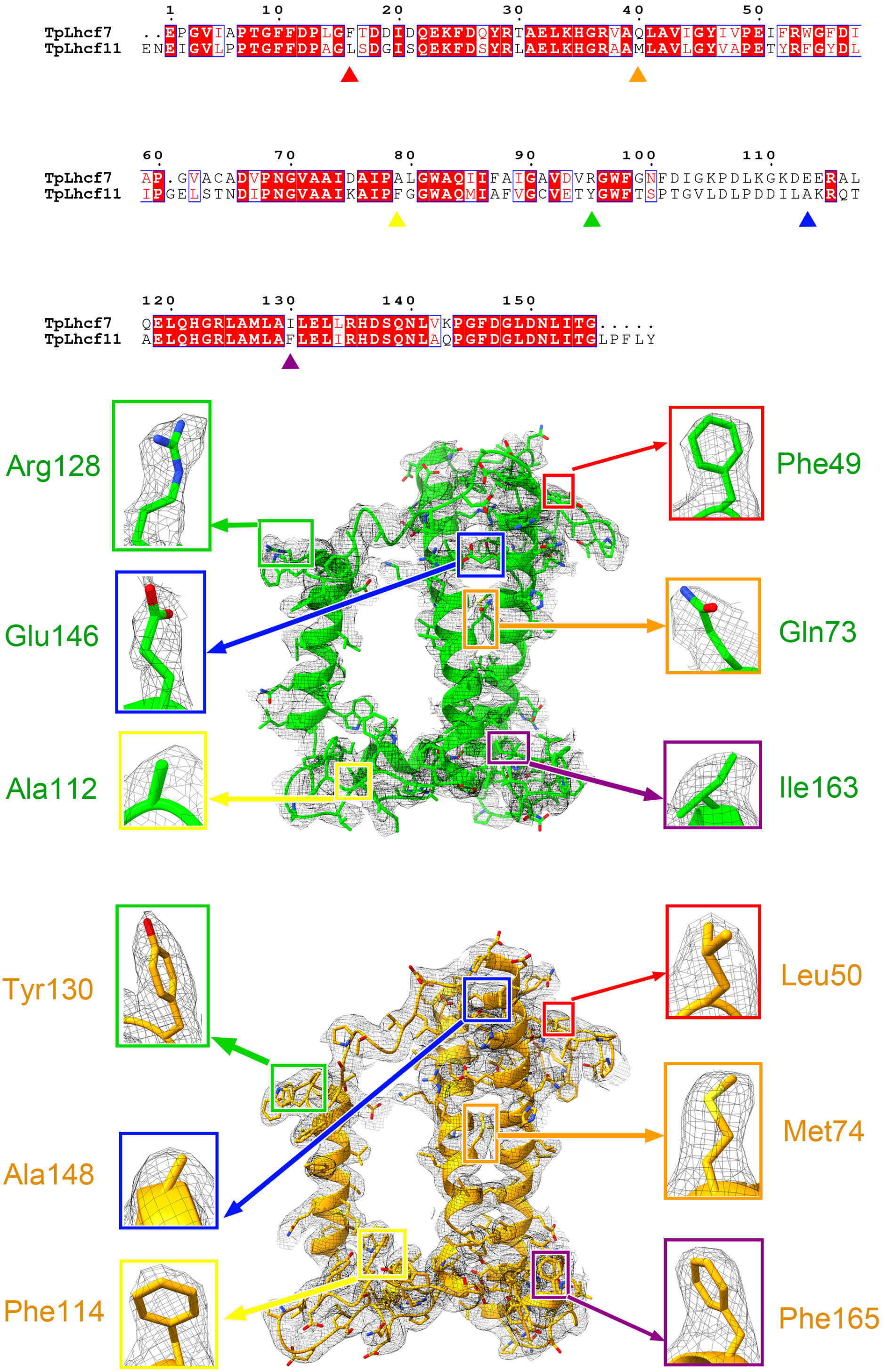

1 10 20 30 40 50
TpLhcf7 ..EPGVIAPTGFFDPLGFTDDIDQEKFDQYRTAELKHGRVAQLAVIGYIVPEIFRWGFDI
TpLhcf11 ENEIGVLPPTGFFDPAGLSDGISQEKFDSYRLAELKHGRAAMLAVLGYVAPETYRFGYDL
60 70 80 90 100 110
TpLhcf7 AP.GVACADVPNGVAAIDAIPALGWAQIIFAIGAVDVRGWFGNFDIGKPDLKGKDEERAL
TpLhcf11 IPGELSTNDIPNGVAAIKAIPFGGWAQMIAFVGCVETYGWFTSPTGVLDLPDDILAKRQT
120 130 140 150
TpLhcf7 QELQHGRLAMLAILELLRHDSQNLVKPGFDGLDNLITG.....
TpLhcf11 AELQHGRLAMLAFLELIRHDSQNLAQPGFDGLDNLITGLPFLY
Arg128
Phe49
Glu146
Gln73
Ala112
Ile163
Tyr130
Leu50
Ala148
Met74
Phe114
Phe165

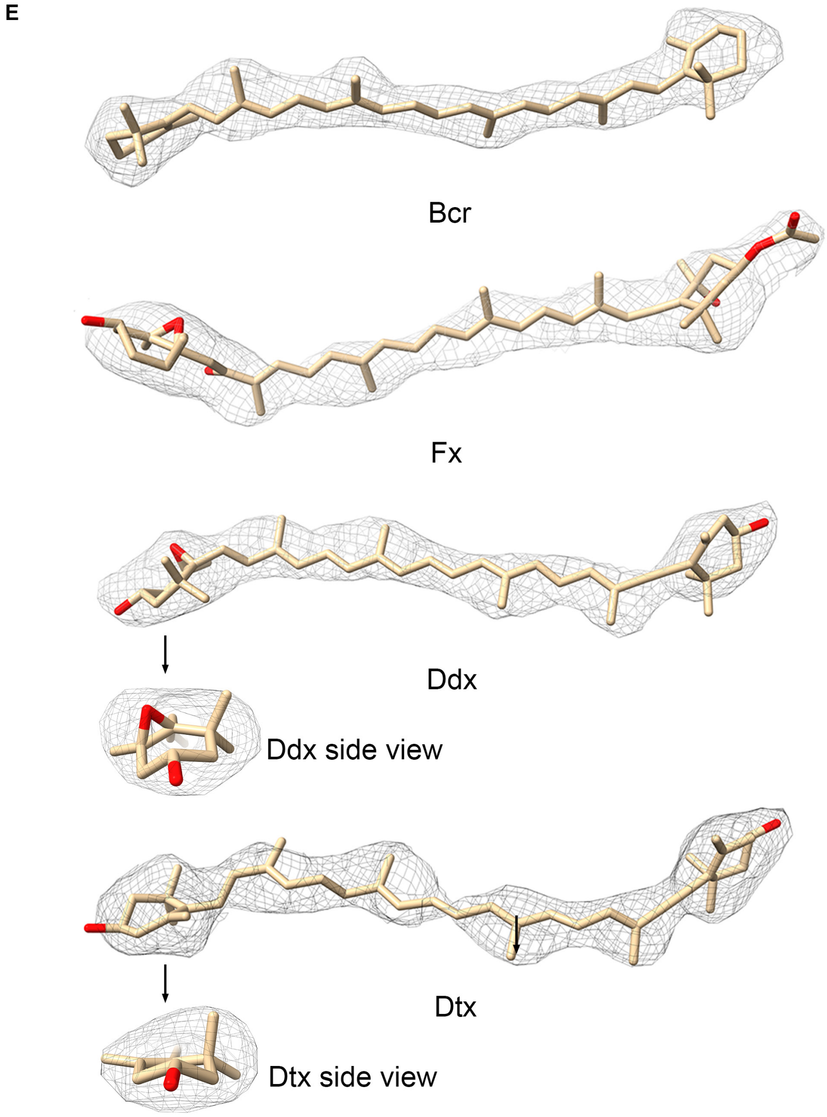

Fig. S3. Examples of cryo-EM density maps for each of the PSII core subunits, antennas, and pigment molecules.

The PSII core and antenna subunits are shown in cartoons with N and O atoms colored blue and red, respectively, and the density maps are shown with a threshold of 0.14 contour level (step 1) by Volume Viewer in Chimera X. The cryo-EM density maps of each subunit and cofactors are depicted in gray meshes. **(A)** Cryo-EM densities and structural models of the PSII core intrinsic and extrinsic subunits. (**B)** Cryo-EM densities of the typical PSII ligands and chlorophyll molecules bound to the PSII-FCPII supercomplex of *T. pseudonana*. (**C)** Cryo-EM density and the structures of six antennas of *T. pseudonana*. The subunits are colored the same as in Fig. 1. Lhcf11/Lhca2/Lhcf6/Lhcf5 are shown with the local maps. (**D)** Two closely related Lhcf7 and Lhcf11 unambiguously fitted in their density map according to their unique sequences. (**E**) Cryo-EM densities of the typical carotenoid molecules bound to the PSII-FCPII supercomplex of *T. pseudonana*.

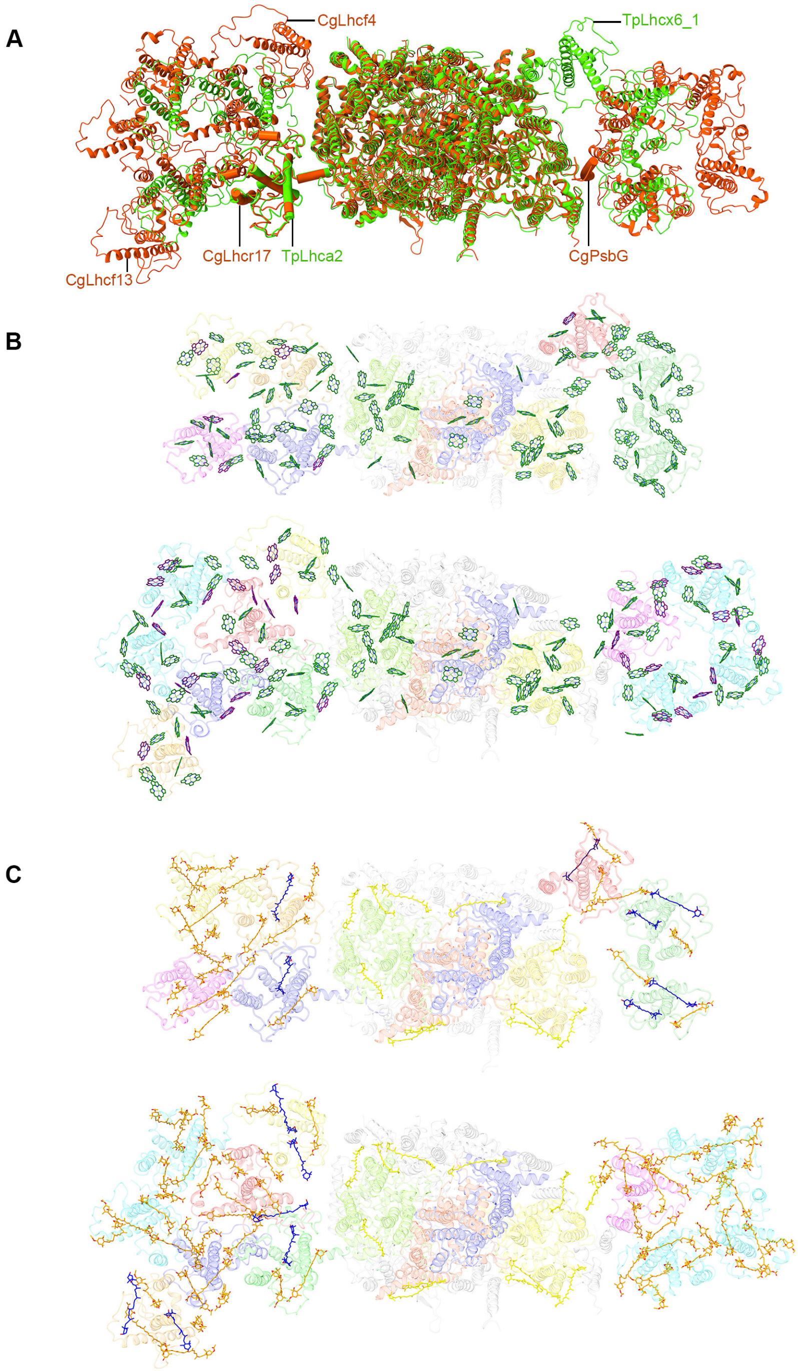

Fig. S4. Comparison of the structures and pigment arrangements between Tp-PSII-FCPII and Cg-PSII-FCP supercomplexes.

(A) Alignment of monomeric Tp-PSII-FCPII (green) and Cg-PSII-FCP (red), among which four linker subunits and one additional Cg-lhcf13 subunit were labeled. (**B and C)** Chlorophyll and carotenoid arrangements of the two monomeric supercomplexes. The upper side of panels B, C is Tp-PSII-FCPII, whereas the lower side is Cg-PSII-FCPII. Chls *a*, Chls *c*, Fx, Ddx, and Dtx molecules are colored forest green, magenta, orange, blue and indigo, respectively.

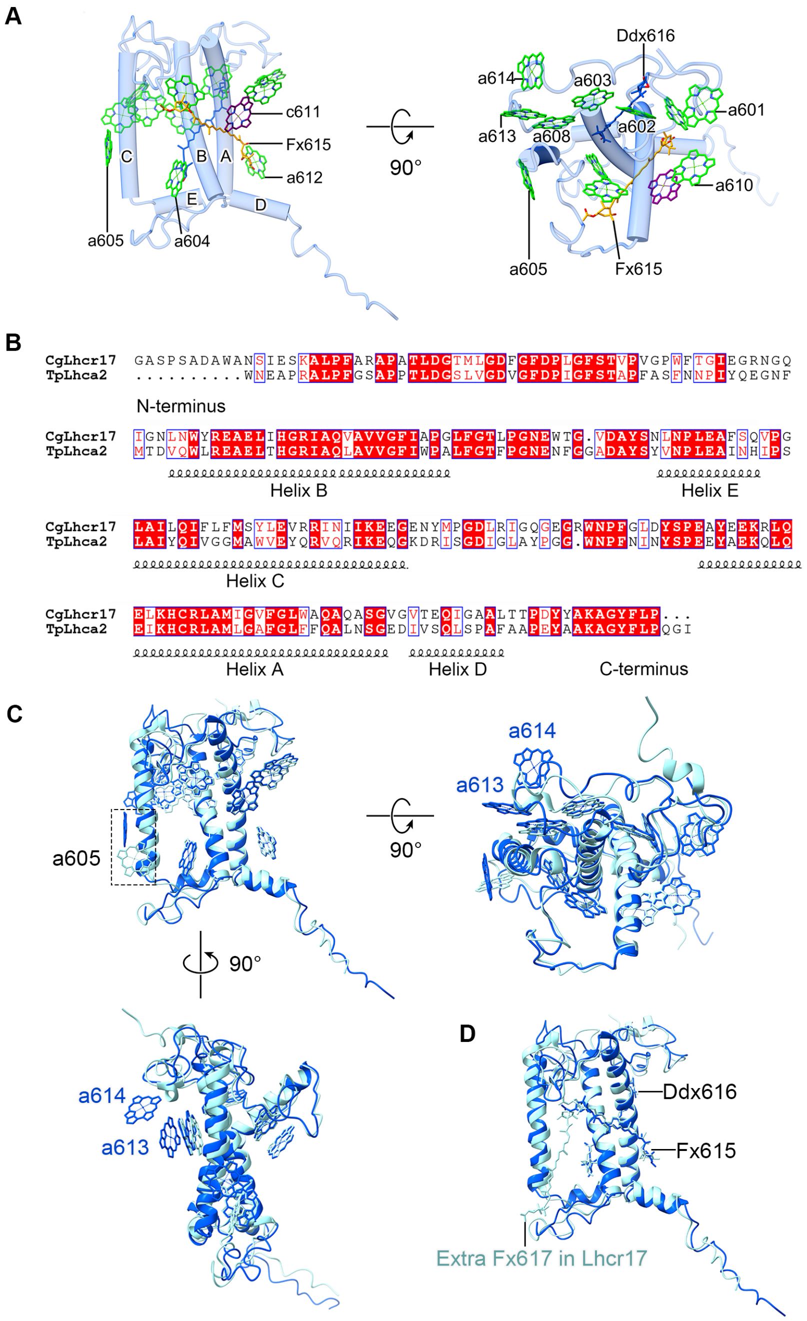

Fig. S5. Structures and pigment arrangements in Tp-Lhca2 and its sequences alignment and structural comparison with Cg-Lhcr17 (Cg-FCP-D).

(A) Structure of Tp-Lhca2. **(B)** Sequences alignment of Tp-Lhca2 and Cg-Lhcr17 (PDB code: 7VD5, Cg-FCP-D). The secondary structures shown below the sequences are taken from the cryo-EM structures. Helices are represented by spiral coils. **(C)** Comparison of the chlorophylls between Tp-Lhca2 (navy) and Cg-Lhcr17 (light blue). Chls 601-604 and 608-612 are conserved in the two structures, whereas additional a613 and a614 are found and the 605 is shifted in Tp-Lhca2; these Chls are labeled. **(D)** Comparison of Fxs between Tp-Lhca2 (navy) and Cg-Lhcr17 (light blue). Fx 615 and Ddx616 sites are conserved, and the Fx617 site in Tp-Lhca2 is lost.

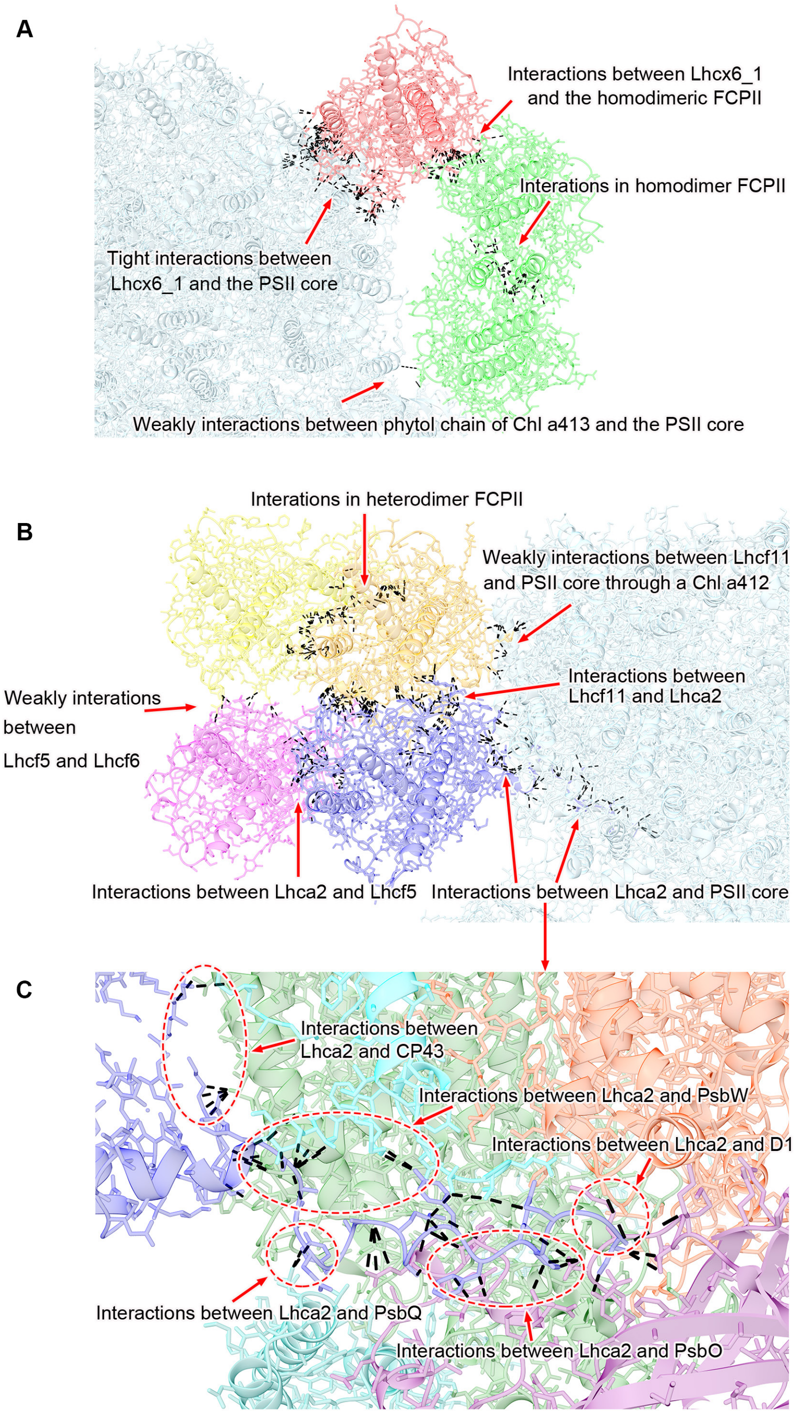

Fig. S6. Van der Waals forces analysis within the adjacent subunits.

(**A**) Homodimeric FCPII connects to the PSII core mainly through Lhcx6_1 (**B**) The connections between the PSII core and Lhcf6/11 as well as Lhcf5 and Lhca2 on one side of the PSII core. Heterodimeric FCPII connects to the PSII core mainly through Lhca2. (**C**) The detailed connections of the long C-terminus of Lhca2 with the PSII core and its interactions with CP43, PsbW, D1, PsbQ, and PsbO at the luminal surface region.

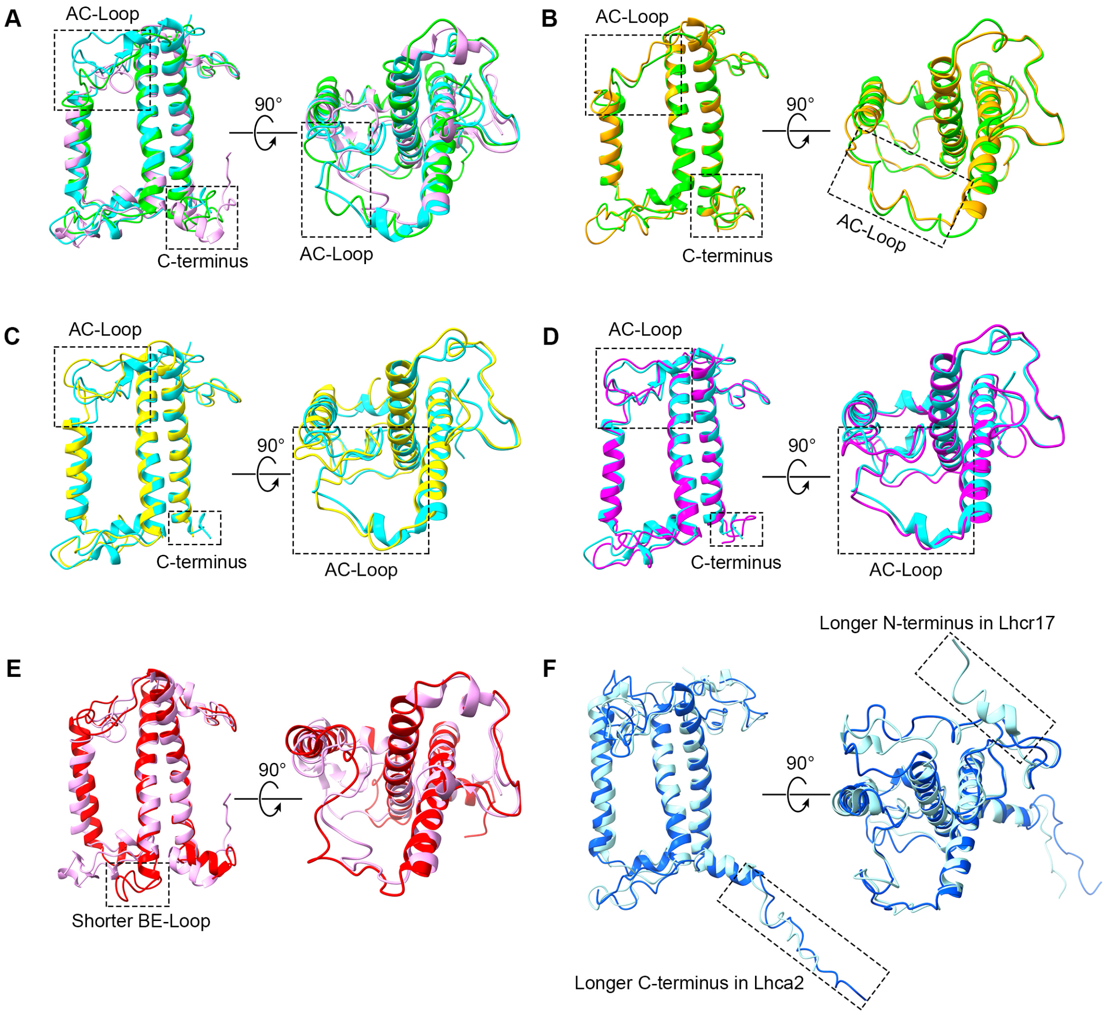

Fig. S7. Comparisons of the typical protein structures among different FCP groups.

(**A**) Comparison of structures of Lhcf7 (green) from *T. pseudonana*, Lhcf1 (light purple) from C. gracilis, and Lhcf4 (cyan) from *P. tricornutum*. (**B**) Comparison of the structures of Lhcf7 (green) and Lhcf11 (orange) from *T. pseudonana*. (**C**) Comparison of the structures of Lhcf6 (green) from T. pseudonana and Lhcf4 (cyan) from *P. tricornutum*. (**D**) Comparison of the structures of Lhcf5 (magenta) from *T. pseudonana* and Lhcf4 (cyan) from *P. tricornutum*. (**E**) Comparison of the structures of Lhcx6_1 (red) from *T. pseudonana* and Lhcf1 (light purple) from *C. gracilis*. (**F**) Comparison of the structures of Lhca2 (navy) from *T. pseudonana* and Lhcr17 (light blue) from *C. gracilis*.

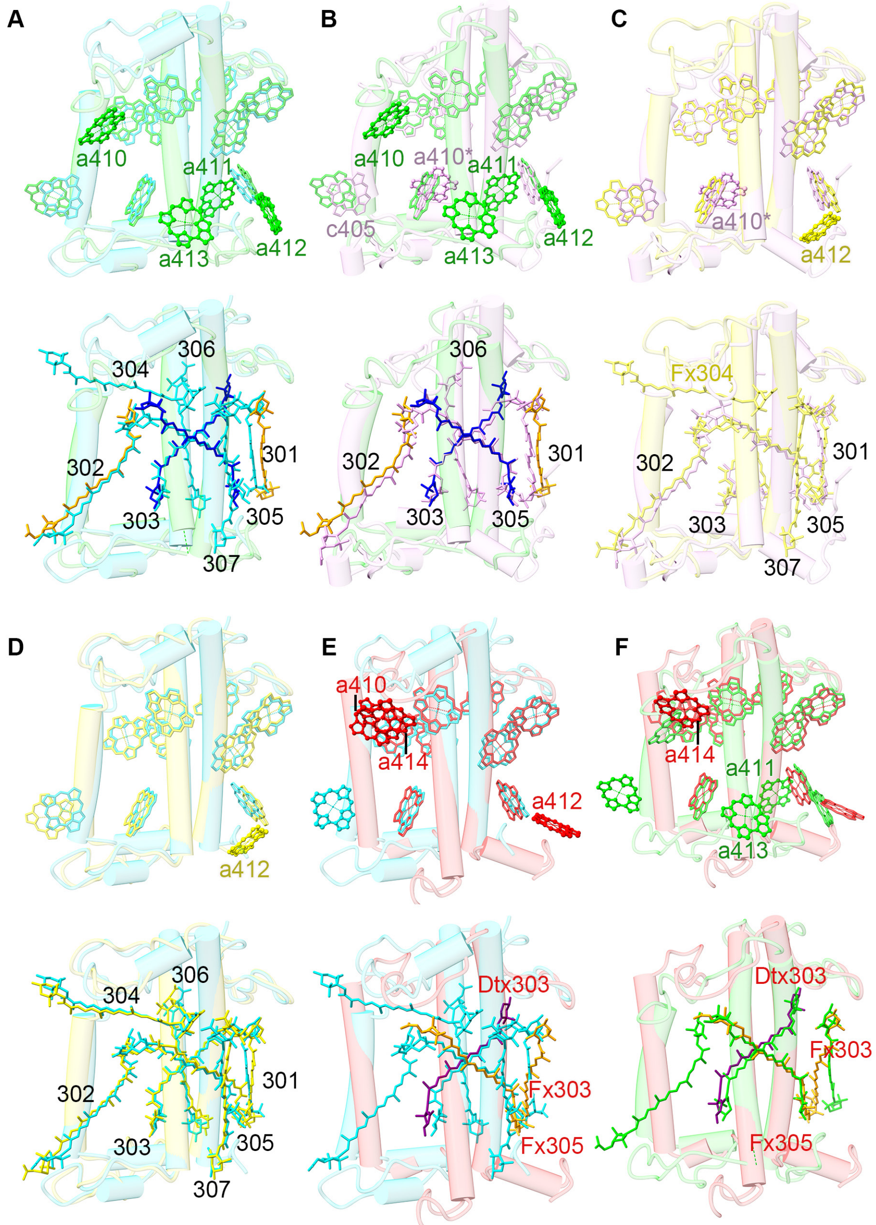

Fig. S8. Comparisons of pigment arrangement in *T. pseudonana* FCPII with other typical FCPs.

(**A**) Alignment of Pt-Lhcf4 (cyan) and Tp-Lhcf7_2 (green). Sites of Chls 401-409 are conserved, and the additional Chls sites in Tp-Lhcf7 are labeled and indicated by ball and sticks with labels. Two Ddx sites in Tp-Lhcf7 and two Fx sites are labeled in blue and orange, respectively. (**B**) Alignment of Cg-Lhcf1 (light purple) and Tp-Lhcf7 (green). A shifted Chl *a*410 and variable Chl *c*405 sites are labeled in Cg-Lhcf1. (**C to D**) Tp-Lhcf6 (yellow) compared with Cg-Lhcf1 (light purple) and Pt-Lhcf4 (cyan). Additional Chl a412 site in Tp-Lhcf6 and additional Chl a410 in Cg-Lhcf1 are labeled and indicated by ball and sticks. Cg-Lhcf1 lacks Fx304. (**E to F**) Tp-Lhcx6_1 compared with Pt-Lhcf4 (cyan) (E) and Tp-Lhcf7 (green) (F). Tp-Lhcx6_1 lacks the Chl a405 site but has additional Chl a412 and Chl a414 sites. Tp-Lhcx6_1 shows a special Dtx303 (indigo) and two conserved Fx303 and Fx305 sites.

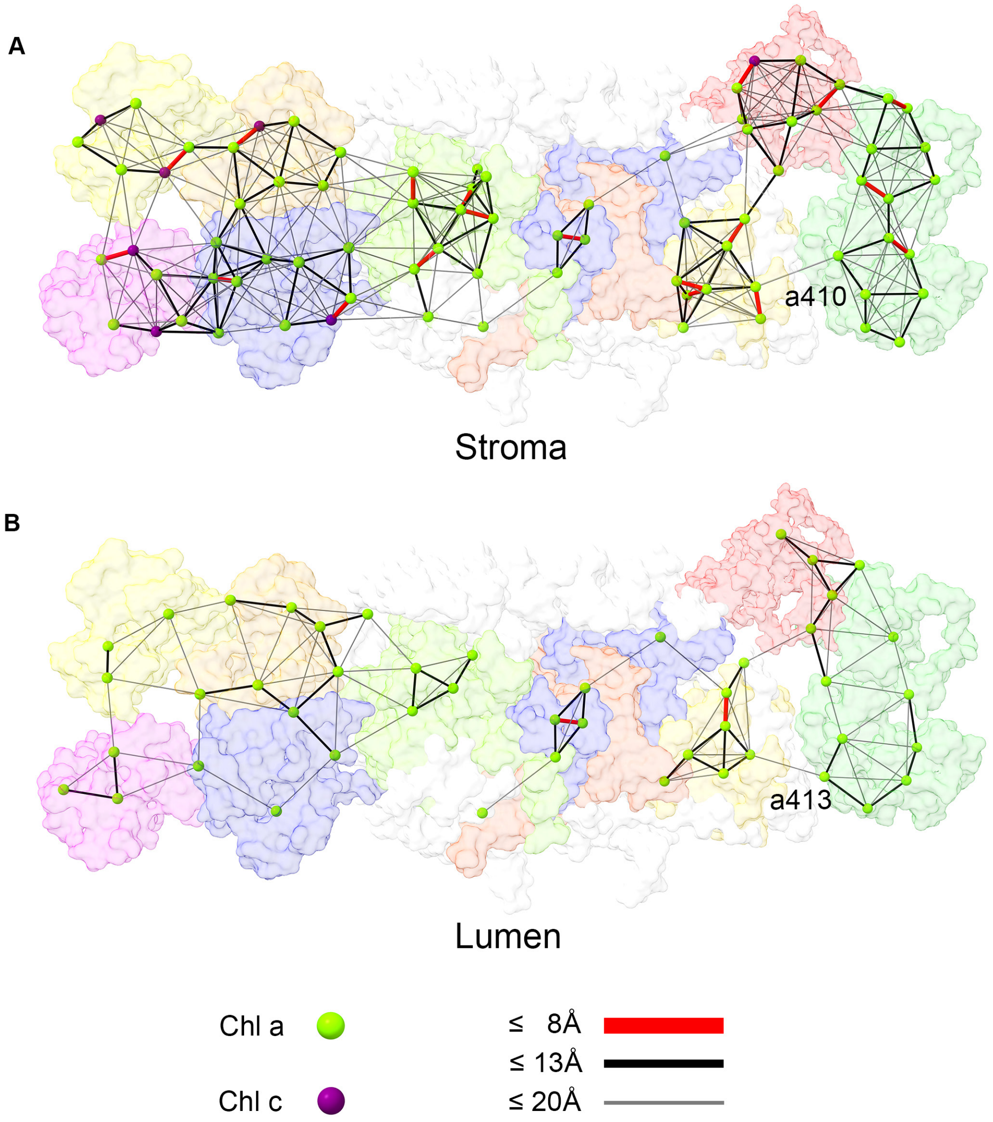

Fig. S9. Network of energy transfer based on distances among Chls.
Distribution of chlorophylls indicated by Mg atoms. Chls *a* and *c* were shown in green and purple, respectively. Pathways with different distances on the stromal **(A)** and lumenal **(B)** sides are connected by different lines, with thicker lines representing shorter distances and therefore more efficient EET.

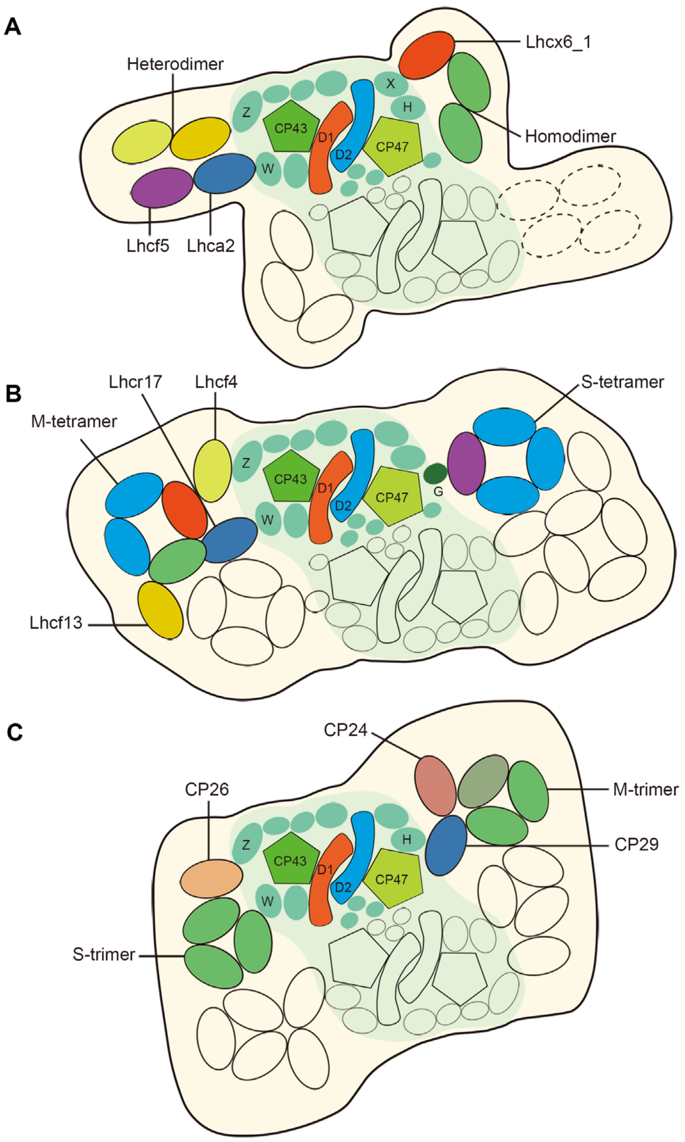

Fig. S10. Structural comparisons between the PSII-LHCII (FCPII) super-complexes from different organisms associated with dimeric, tetrameric or trimeric antennas.

(A) Tp-PSII-FCPII supercomplex revealed by this study (PDB code: 8IWH). **(B)** PSII-FCPII supercomplex from a diatom *C. gracilis* (PDB code: 7VD5). **(C)** PSII-LHCII supercomplex from *P. sativum* (PDB code: 5XNL).

Table S1. Mass spectrometry for PSII-FCPII in Fig. 1B

Source data 1. Mass spectrometric data of gel 1 separated by SDS-PAGE.

Identification	UniProt ID	Score	Coverage
PsbO	B8C4I5	303.41	78.36
CP43	A0T096	277.02	24.63
CP47	A0T0P5	202.72	30.06
D2	A0T0T0	121.26	20.51
D1	A0T0W2	154.33	17.22

Source data 2. Mass spectrometric data of gel 2 separated by SDS-PAGE.

Identification	UniProt ID	Score	Coverage
PsbV	B8LES1	121.74	53.99
PsbQ	B8BSY9	79.50	24.64
Lhcx6_1	B5YLU3	143.94	41.21

Source data 3. Mass spectrometric data of gel 3 separated by SDS-PAGE.

Identification	UniProt ID	Score	Coverage
PsbU	B8BVI4	74.96	26.35
Psb31	B8CC14	56.34	18.10
PsbE	B8LEU5	30.36	9.50

Source data 4. Mass spectrometric data of Lhcf7 separated by SDS-PAGE.

Identification	UniProt ID	Score	Coverage
Lhcf7	B8BX93	295.68	60.82

Source data 5. Mass spectrometric data of Lhcf11 separated by SDS-PAGE.

Identification	UniProt ID	Score	Coverage
Lhcf11	B8BVI1	153.76	43.88

Source data 6. Mass spectrometric data of Lhcf6 separated by SDS-PAGE.

Identification	UniProt ID	Score	Coverage
Lhcf6	B8BX92	171.75	66.67

Source data 7. Mass spectrometric data of Lhcf5 separated by SDS-PAGE.

Identification	UniProt ID	Score	Coverage
Lhcf5	B8CEV5	160.08	53.50
Lhcf1	B8CFW3	97.34	40.00

Source data 8. Mass spectrometric data of Lhca2 separated by SDS-PAGE.

Identification	UniProt ID	Score	Coverage
Lhca2	B5YLU3	126.63	42.00

The bands on the gel were cut into 8 groups and identified by mass spectrometry, respectively.

Band gel 1: typical large PSII core subunits, PsbO, CP47, CP43, D2, D1; Band gel 2: Lhcx6_1 FCP antenna and PsbQ and PsbV core subunits; Band gel 3: some PSII core subunits, PsbU, Psb31, PsbE; Band gel 4: Lhcf7 FCP antenna; Band gel 5: Lhcf11 FCP antenna; Band gel 6: Lhcf6 FCP antenna; Band gel 7: Lhcf5 and Lhcf1 FCP antennas; Band gel 8: Lhca2 FCP antenna.

Score: The sum of the score in the search results for the peptides identified to that protein.

Coverage: Proportion of the number of amino acids identified by the protein in mass spectrometry analysis to the total number of amino acids of the highest-scored protein in the proteome.

Table S2. The pigment composition of the PSII-FCPII samples analyzed by HPLC and the modeled pigments in the complex

Samples	Chl a/c	Chl a	Chl c	β-carotene	Fx	Ddx	Dtx
PSII-FCPII in low light	24.4	100.0±0.42	4.1±0.08	12.0±0.32	16.9±0.44	5.9±0.19	1.7±0.17
PSII-FCPII in high light	27.8	100.0±0.47	3.6±0.11	11.6±0.26	16.1±0.63	3.9±0.26	3.3±0.22
PSII-FCPII in modelled	22.9	100	4.4	12.0	15.8	5.5	1.1

Values shown in the table are the average of 3 measurements with the standard deviations, indicated as the composition and ratio of either pigments per 100 Chl a, the PSII-FCPII in low light or high light were normalized by the content of chlorophyll (Chl) a.

Table S3. Cryo-EM data collection, refinement, and validation statistics.

**Model statistics**	**PSII-FCPII**	**Four FCPIIs**
PDB code<br>EMDB ID	8IWH<br>EMD-35766	8J0D<br>EMD-35899
**Data collection and processing**		
Magnification	81000×	81000×
Voltage (kV)	300	300
Electron exposure (e^{-}/Å2)	60	60
Defocus range (μm)	-1.0 ~ -2.0	-1.0 ~ -2.0
Pixel size (Å)	0.44	0.44
Symmetry imposed	C1	C1
Initial particle images (no.)	787,586	787,586
Final particle images (no.)	97,098	97,098
Map resolution (Å)	2.68	3.19
FSC threshold	0.143	0.143
**Refinement**		
Number of atoms	75529	9105
Protein residues	7531	711
Ligands	407	74
R.m.s. deviations		
Bond lengths (Å)	0.004	0.006
Bond angles (°)	0.798	0.971
Validation		
MolProbity score	1.76	1.98
Clashscore	7.44	10.63
Rotamer outliers (%)	0.00	0.96
Ramachandran plot		
Favored (%)	94.87	93.25
Allowed (%)	4.76	6.01
Outliers (%)	0.38	0.74

Table S4. Lengths of amino acid residues and cofactors constructed in the *T. pseudonana* PSII-FCPII structure.

Subunits	Chain names	*Source (UniProt ID)*	Residues	Chls	Cars	Lipids and ligands
PsbA (D1)	A, a	A0T0W2	11-343	4 Chls *a*, 2 Pheophytins	2 Bcrs	1 Mn_4CaO_5, 1 LMG 2 SQDGs, 1 Fe, 1 Cl^-
PsbB (CP47)	B, b	A0T0P5	2-479	16 Chls *a*	3 Bcrs	4 LMGs, 2 SQDGs, 1 DGDG
PsbC (CP43)	C, c	A0T0T1	21-470	13 Chls *a*	4 Bcrs	2 LMG, 1 LHG, 3 DGDGs
PsbD (D2)	D, d	A0T0T0	11-351	2 Chls *a*	1 Bcr	1 Bicarbonate ion, 1 LMG, 3 LHG, 1 Plastoquinone
PsbE	E, e	B8LEU5	8-83			
PsbF	F, f	A0T0U1	13-43			1 Heme
PsbH	H, h	A0T0P8	2-66		1 Bcr	1 DGDG
PsbI	I, i	A0T0U9	1-34			1 SQDG
PsbJ	J, j	A0T0T9	6-38			
PsbK	K, k	A0T0S8	8-44			
PsbL	L, l	A0T0U0	2-38			1 LHG
PsbM	M, m	B8BYH2	72-112			
Unknown1	N, n	Unknown	Poly-alanines			
PsbO	O, o	B8C4I5	58-305			
PsbQ	Q, q	B8BSY9	56-211			
PsbT	T, t	A0T0P6	1-28			
PsbU	U, u	B8BVI4	56-147			
PsbV	V, v	A0T0N2	28-162			1 Heme
PsbW	W, w	B8C8Y3	178-231	1 Chl *a*		
PsbX	X, x	A0T0N4	1-35			
PsbY	Y, y	A0T0V3	1-34			
PsbZ	Z, z	A0T0T3	1-61	1 Chl *a*		2 LMG
Psb31	G, g	B8CC14	57-176			
Lhcx6_1	0, 7	B5YLU3	35-199	10 Chls *a*, 1 Chl *c*	2 Fxs, 1 Dtx	3 LHG, 1 LMG, 1 SQDGs
Lhca2	3	B8BUU4	31-250	11 Chls *a*, 1 Chl *c*	1Fx, 1 Ddx	2 LHGs, 2 LMGs
Lhcf5	6	B8CEV5	32-199	8 Chls *a*, 2 Chl *c*	6 Fxs	
Lhcf7_1	1, 8	B8BX93	31-193	12 Chls *a*,	2 Fxs, 2 Ddxs	
Lhcf7_2	2, 9	B8BX93	31-193	13 Chls *a*	2 Fxs, 2 Ddxs	
Lhcf11	4	B8BVI1	33-195	12 Chls *a*, 1 Chl *c*	3 Fxs, 1 Ddx	1 LHG, 1 DGDG
Lhcf6	5	B8BX92	33-192	8 Chls *a*, 2 Chls *c*	7 Fxs	1 LHG, 1 LMG
Total				138 Chls *a*, 8 Chls *c*, 2 Pheophytins	29 Fx, 10 Ddx, 2 Dtx, 22 BCR	20 LHGs, 26 LMGs, 11 DGDGs, 12 SQDGs, 2 HCO_3^-, 4 Hemes, 2 Plastoquinones, 2 Fe, 2Cl^-, 2 Mn_4CaO_5

Chl: chlorophyll; **Fx:** fucoxanthin; **BCR:** β-carotene; **Ddx:** diadinoxanthin; **Dtx:** diatoxanthin;

LHG: 1,2-Dipalmitoyl-phosphatidyl-glycerole; **LMG**: 1,2-distearoyl-monogalactosyl-diglyceride; **SQDG**: 1,2-di-O-acyl-3-O-[6-deoxy-6-sulfo-alpha-D-glucopyranosyl]-Sn-glycerol; **DGDG**: digalactosyl diacyl glycerol.

Table S5. Sequence identities and RMSD values among different FCP antennas from different species of diatoms.

Protein	Identity (%) with Lhcf7 (*Tp*)	Identity (%) with Lhcf4 (*Pt*)	Identity (%) with Lhcf1 (*Cg*)	RMSD (Å) / Cα of Lhcf7 (*Tp*)	RMSD (Å) /Cα of Lhcf4 (*Pt*)	RMSD (Å) / Cα of Lhcf1 (*Cg*)
Lhcf7_1 (*Tp*)	100	37	31	0	1.10/148	1.11/155
Lhcf7_2 (*Tp*)	100	37	31	0.44/162	1.09/147	1.14/155
Lhcf11 (*Tp*)	60	33	29	0.92/162	1.08/144	1.06/155
Lhcf6 (*Tp*)	39	59	40	1.14/141	0.94/157	0.89/149
Lhcf5 (*Tp*)	37	62	40	1.08/151	0.81/164	1.02/161
Lhcf4 (*Pt*)	37	100	37	1.10/148	0	0.95/157
Lhcf1 (*Cg*)	31	37	100	1.11/155	0.95/157	0
Lhcx6_1 (*Tp*)	29	28	28	0.91/141	0.99/144	0.96/154

The identity between Lhca2 (*Tp*) and Lhcr17 (*Cg*) is 51%, and the RMSD between Lhca2 (*Tp*)and Lhcr17 (*Cg*) is 1.10/216 (Å) / Cα

RMSD: Root means square deviation values between two secondary structures of subunits are calculated by Chimera. Identity between the amino acid sequence of Lhcf7 (*T. pseudonana*) and those of Lhcf4 (*P. tricornutum*), Lhcf1 (*C. gracilis*) were estimated by an optimal global alignment of two sequences using EMBOSS needle (https://www.ebi.ac.uk/Tools/psa/emboss_needle/) with the BLOSUM62 matrix and default settings.

Table S6. Pigment-binding sites in the FCP antennas from *T. pseudonana* were revealed in the present study.

**Pigments**	**Lhcf7_1 (*Tp*)**	**Lhcf7_2 (*Tp*)**	**Lhcf11 (*Tp*)**	**Lhcf5 (*Tp*)**	**Lhcf6 (*Tp*)**	**Lhcf4 (*Pt*)**	**Lhcf1 (*Cg*)**	**Lhcx6_1 (*Tp*)**
401	*a*	*a*	*a*	*a*	*a*	*a*	*a*	*a*
402	*a*	*a*	*a*	*a*	*a*	*a*	*a*	*a*
403	*a*	*a*	*c*-	*c*	*c*	*c*	*c*	*c*
404	*a*	*a*	*a*	*a*	*a*	*a*	*a*	*a*
405	*a*	*a*	*a*	*a*	*a*	*a*	*c*	-
406	*a*	*a*	*a*	*a*	*a*	*a*	*c*	*a*
407	*a*	*a*	*a*	*a*	*a*	*a*	*a*	*a*
408	*a*	*a*	*a*	*c*	*c*	*c*	*c*	*a*
409	*a*	*a*	*a*	*a*	*a*	*a*	*a*	*a*
410	*a*	*a*	*a*	*a**	-	-	*a**	*a*
411	*a*	*a*	*a*	-	-	-	-	-
412	*a*	*a*	*a*	-	*a*	-	-	*a*
413	-	*a*	*a*	-	-	-	-	-
414	-	-	-	-	-	-	-	*a*
301	Fx	Fx	Fx	Fx	Fx	Fx	Fx	Fx
302	Fx	Fx	Fx	Fx-	Fx	Fx	Fx	-
303	Ddx	Ddx	Ddx	Fx	Fx	Fx	Fx	Dtx
304	-	-	-	-	Fx	Fx	-	-
305	Ddx	Ddx	Fx	Fx	Fx	Fx	Fx	Fx
306	-	-	-	Fx	Fx	Fx	Fx	-
307	-	-	-	Fx-	Fx	Fx	Fx	-
308	-	-	-	-	-	Ddx	-	-

*a**: The Chl a has the same conserved amino acid site, but the pigment site is shifted;;

c-: The density map for the site of Chl c is less certain;

Fx-: The density map for the sites of Fxs is less certain.

Table S7. Pigment-binding sites in Lhca-like antennas.

**Pigments**	**Lhca2 (*Tp*)**	**Lhcr17 (*Cg*)**	**Lhca2 (*Ps*)**
601	*a*	*a*	*b*
602	*a*	*a*	*a*
603	*a*	*a*	*a*
604	*a*	*a*	*a*
605	*a*	*a*	*b*
606	-	-	*b*
607	-	-	*b*
608	*a*	*a*	*a*
609	*a*	*a*	*a*
610	*a*	*a*	*a*
611	*c*	*c*	*a*
612	*a*	*a*	*a*
613	*a**	-	*a*
614	*a**	-	*b*
615	Fx	Fx	Lut
616	Ddx	Ddx	Xat
617	-	Fx	Bcr

*a**:The Chl a has the same conserved amino acid site, but the pigment site is shifted;

Table S8. Chlorophyll *c*-binding sites in Tp-PSII-FCPII, and the sites of surrounding alkaline amino acids and Fxs that interact with polar C-17 propionic acid of Chl *c*.

Chlorophylls c	Antenna subunit	Central ligands
Chl *c*403	Lhcx6_1	Arg61
Chl *c*611	Lhca2	Lys196
Chl *c*403	Lhcf11	Arg133
Chl *c*403	Lhcf6	Arg61
Chl *c*408	Lhcf6	Fx307
Chl *c*403	Lhcf5	Arg61
Chl *c*408	Lhcf5	Lys166

Source data 1-8. Mass spectrometric data results of SDS-PAGE gels of PSII-FCPII

Source data 1. Mass spectrometric data of gel 1 (including typical large PSII core subunits) separated by SDS-PAGE.

Source data 2. Mass spectrometric data of gel 2 (including Lhcx6_1 FCP antenna and PsbQ and PsbV core subunits) separated by SDS-PAGE.

Source data 3. Mass spectrometric data of gel 3 (including some PSII core subunits, PsbU, Psb31, PsbE) separated by SDS-PAGE.

Source data 4. Mass spectrometric data of Lhcf7 separated by SDS-PAGE.

Source data 5. Mass spectrometric data of Lhcf11 separated by SDS-PAGE.

Source data 6. Mass spectrometric data of Lhcf6 separated by SDS-PAGE.

Source data 7. Mass spectrometric data of Lhcf5 separated by SDS-PAGE.

Source data 8. Mass spectrometric data of Lhca2 separated by SDS-PAGE.
